# Unrestrained Gα_i2_ Signaling Disrupts Neutrophil Trafficking, Aging, and Clearance

**DOI:** 10.3389/fimmu.2021.679856

**Published:** 2021-05-31

**Authors:** Serena Li-Sue Yan, Il-Young Hwang, Olena Kamenyeva, Juraj Kabat, Ji Sung Kim, Chung Park, John H. Kehrl

**Affiliations:** ^1^ Laboratory of Immunoregulation, National Institute of Allergy and Infectious Diseases, National Institutes of Health, Bethesda, MD, United States; ^2^ Biological Imaging, Research Technology Branch, National Institute of Allergy and Infectious Diseases, National Institutes of Health, Bethesda, MD, United States

**Keywords:** neutrophil, chemotaxis, trafficking, chemoattractants, G-protein, intravital, RGS protein

## Abstract

Neutrophil trafficking, homeostatic and pathogen elicited, depends upon chemoattractant receptors triggering heterotrimeric G-protein Gα_i_β*γ* signaling, whose magnitude and kinetics are governed by RGS protein/Gα_i_ interactions. RGS proteins typically limit Gα_i_ signaling by reducing the duration that Gα_i_ subunits remain GTP bound and able to activate downstream effectors. Yet how in totality RGS proteins shape neutrophil chemoattractant receptor activated responses remains unclear. Here, we show that C57Bl/6 mouse neutrophils containing a genomic knock-in of a mutation that disables all RGS protein-Gα_i2_ interactions (G184S) cannot properly balance chemoattractant receptor signaling, nor appropriately respond to inflammatory insults. Mutant neutrophils accumulate in mouse bone marrow, spleen, lung, and liver; despite neutropenia and an intrinsic inability to properly mobilize from the bone marrow. *In vitro* they rapidly adhere to ICAM-1 coated plates, but *in vivo* they poorly adhere to blood vessel endothelium. Those few neutrophils that cross blood vessels and enter tissues migrate haphazardly. Following Concanavalin-A administration fragmented G184S neutrophils accumulate in liver sinusoids leading to thrombo-inflammation and perivasculitis. Thus, neutrophil Gα_i2_/RGS protein interactions both limit and facilitate Gα_i2_ signaling thereby promoting normal neutrophil trafficking, aging, and clearance.

## Introduction

Signaling *via* the chemokine receptors CXCR2 and CXCR4 plays several essential roles in neutrophil trafficking. CXCR2 and CXCR4 counter regulate the release of mature neutrophils from the bone marrow (BM) into the circulation (1). Either excessive CXCR4 signaling or a lack of CXCR2 signaling causes myelokathexis (1, 2). Myelokathexis is the inappropriate retention (kathexis) of neutrophils (myelo) in the BM. CXCR2 signaling also triggers diurnal changes in the transcriptional and migratory properties of circulating neutrophils, a process termed neutrophil aging (3, 4). Bmal1 [brain and muscle aryl hydrocarbon receptor nuclear translocator (ARNT)-like 1, encoded by *Arntl*] regulated expression of CXCL2 triggers these CXCR2-dependent changes in circulating neutrophils, which are opposed by CXCR4 signaling. Neutrophils released from the BM express high levels of CD62L that progressively decline during the day, while increasing amounts of surface CXCR4 promotes neutrophil egress from the blood into the tissues, which leads to their clearance by tissue macrophages (3, 5). Deletion of CXCR2 from neutrophils prevents phenotypic aging, whereas deletion of CXCR4 promotes unrestrained aging (3). Neutrophil aging impairs CD62L mediated endothelial rolling, thereby reducing neutrophil accumulation at sites of inflammation (3). Yet aged neutrophils can efficiently cross the endothelium to enter tissues. The removal of aging neutrophils from the circulation allows their clearance, helping to protect blood vessels from potential neutrophil mediated insults (3, 6).

Both CXCR2 and CXCR4 as well as other chemoattractant receptors that help control leukocyte and neutrophil recruitment and trafficking use the heterotrimeric G-protein Gα_i_β*γ* to link to downstream effector molecules (1, 7, 8). Ligand engagement of chemoattractant receptors trigger a conformational change that facilitates receptor/heterotrimeric G-protein coupling, Gαi subunit GDP-GTP exchange, functional Gαi dissociation from Gβ*γ* subunits, downstream effector activation leading to integrin activation, cell polarization, and directed migration (9, 10). Since Gαi subunits possess an intrinsic GTPase activity, GTP hydrolysis facilitates re-assembly of the heterotrimeric G-protein causing signaling to cease (9, 11). By dramatically accelerating the intrinsic GTPase activity of Gαi subunits, RGS proteins reduce the duration that Gαi subunits remains GTP bound, thereby decreasing effector activation by reducing available Gαi-GTP and free Gβγ (11).

Despite the importance of G_i_-coupled receptors in neutrophil trafficking and aging, relatively little is known about the overall importance of RGS proteins in chemoattractant receptor signaling. Murine neutrophils prominently express *Rgs2*, *Rgs18*, and *Rgs19;* lesser amounts of *Rgs3* and *Rgs14;* and detectable levels of mRNA transcripts for several other RGS proteins (http://www.immgen.org/databrowser/index.html). They also highly express *Gnai2*, with a lower amount of *Gnai3* (approximately 1/5 the amount as assessed by RNA sequencing) and little or no *Gnai1* or *Gnao*. Loss of *Rgs2* in mice increases neutrophil recruitment to inflamed lungs (12, 13). Despite its low expression level, loss of *Rgs5* in mice also leads to a more robust recruitment of neutrophils to inflamed lungs (14). In addition, purified neutrophils from these mice had exaggerated responses to CXCR2 and CXCR4 ligands (14). Since the loss of an individual RGS proteins often causes a relatively mild phenotype, perhaps a consequence of their redundant expression profiles, we have made use of a genetically modified mouse that has a mutation, which replaces glycine 184 in the Gα_i2_ protein with a serine (15, 16). This change blocks the binding of RGS proteins to Gα_i2_, thereby rendering RGS proteins unable to act as GTPase activating proteins (GAPs). We will refer to mice or neutrophils bearing the Gα_i2_ G184S mutation on both alleles as G184S mice or neutrophils, respectively. Previously, we have shown that the G184S mice accumulate BM neutrophils, which mobilize poorly to an inflamed peritoneum or in response to sterile ear inflammation. Also, these mice did not control a normally nonlethal *Staphylococcus aureus* infection (17).

In this study we have further investigated the trafficking patterns and mobilization of G184S neutrophils to inflammatory stimuli predominately using mice reconstituted with either WT or G184S BM, or with a 1:1 mixture. Our studies indicate the G184S Gα_i2_ mutation causes an initial misbalance in the BM between the CXCR4 mediated retention signal and the CXCR2 mediated mobilization signal. Those neutrophils that escape the BM rapidly leave the circulation to accumulate in the marginal pools located in the lung, liver, and spleen. Neutrophil recruitment to inflammatory sites is severely impaired secondary to both a mobilization defect and to impaired transendothelial migration (TEM). Furthermore, G184S BM reconstituted mice poorly tolerate ConA induced inflammation as G184S neutrophils fragment and aggregate in liver sinusoids. The significance of these results is discussed.

## Material And Methods

### Mice

Male (20-25g) mice were used. C57BL/6 and B6.SJL-Ptprc^a^ Pepc^b^/BoyJ mice were obtained from the Jackson Laboratory. B6.129S1-*Gnai2^tm1.1Rneu^*/J (G184S), Jackson Laboratory mice were backcrossed >17 times onto C57BL/6. Litter mate controls were used for experiments that directly compared wild-type (WT) and G184S mice. The G184S mice were bred as heterozygotes. All mice were maintained in specific-pathogen-free conditions at an Association for Assessment and Accreditation of Laboratory Animal Care-accredited animal facility at the NIAID. All procedures and protocols regarding animal experiments were performed under a study protocol approved by the NIAID Animal Care and Use Committee (National Institutes of Health). For the BM reconstitutions, 6 wk-old C57BL/6 (CD45.1) mice were irradiated twice with 550 rad for total of 1100 rad and received BM from C57BL/6 CD45.2 mice (control) or from G184S CD45.2 mice. Mixed chimeric mice were made by reconstituting C57BL/6 CD45.1 mice with a 1:1 mix of BM from C57BL/6 CD45.2 mice (WT) and from G184S CD45.2 mice. The engraftment was monitored by sampling the blood 28 d later. The mice were used 6 – 7 weeks after reconstitution. All mice used in this study were 6 – 12 weeks of age.

### Reagents

A resource table is included in the supplement file, which describes and lists the sources of the antibodies, chemicals, peptides, recombinant proteins, assays, and software used in this study.

### Cell Isolation and Preparation

Spleens and bone marrow cells were mechanically disrupted by their passage through a syringe and single-cell suspensions were obtained by filtration through cell strainers (75 μ m; BD Biosciences). For comparison purposes when we isolated cells from individual wild type and G184S bone marrow reconstituted mice we isolated all the cells from spleen, a single femurs, or from both lungs. 50ul of whole blood was collected from the mandibular plexus or the tail vein. BM and splenic neutrophils were purified to a purity of ~95% using anti-Ly-6G MicroBeads UltraPure (Miltenyi Biotech). Preparations of lung single cells was based on a previously describe method (18). When needed neutrophils were cultured in RPMI 1640 containing 10% fetal calf serum (FCS, Gibco), 2 mM L-glutamine, antibiotics (100 IU/mL penicillin and 100 μg/mL streptomycin), 1 mM sodium pyruvate, and 50 µM 2-mercaptoethanol. Cell culture media for S1P chemotaxis was same as above except charcoal-dextran filtered FCS serum was used.

### Flow Cytometry

Single cells were re-suspended in PBS, 2% FCS, and stained with fluorochrome-conjugated or biotinylated antibodies against CD11b (M1/70), Ly6G (1A8), Ly6C (AL-21), CD11c (HL3), F4/80 (BM8), NK1.1 (PK136), TCR*γ*δ (GL3), B220 (RA3-6B2), CD117 (2B8), TER-119 (TER-119), CD3 (145-2C11), CD4 (GK1.5), CD5 (53-7.3), CD8 (53-6.7), CD184 (CXCR4, 2B11), CXCR2 (SA044E1), CD62L (MEL-16), CD45.1 (A20), or CD45.2 (104) (all from Thermo Fisher, BioLegend, or BD Pharmingen). Biotin-labeled antibodies were visualized with fluorochrome-conjugated streptavidin (BioLegend). LIVE/DEAD™ Fixable Aqua Dead Cell Stain Kit, LIVE/DEAD™ Fixable Near-IR Dead Cell Stain Kit or LIVE/DEAD™ Fixable Yellow Dead Cell Stain Kit (Thermo Fisher) were used in all experiments to exclude dead cells. Compensation was performed using AbC™ Total Antibody Compensation Bead Kit (Thermo Fisher) and ArCTM Amine Reactive Compensation Bead (Thermo Fisher) individually stained with each fluorochrome. Compensation matrices were calculated with FACSdiva software. Data acquisition included cell number count was done on MACSQuant, FACSCanto II and FACSCelesta SORP (BD) flow cytometer and analyzed with FlowJo 9.9 and 10.7 software (Treestar). Dead cells were gated out by staining with Live/Dead Fixable dye during data collection. For intracellular flow cytometry cells were intracellular stained using the eBioscience™ Intracellular fixation & permeabilization buffer Set (Thermo Fisher) protocol using the PE conjugated CXCR2, PE-conjugated CXCR4 antibody, phallodin, or pERM antibody. To detect phosphorylated ERM proteins a rabbit anti-phospho–ezrin (Thr^567^)/radixin (Thr^564^)/moesin (Thr^558^) (pERM) antibody was used (Cell Signaling Technology). Isotype control staining was performed using rabbit IgG isotype mAb Alexa Fluor 647 (DA1E; Cell Signaling Technology). Secondary F(ab′)_2_ fragment of goat anti-rabbit IgG (H+L) Alexa Fluor 647 (Thermo Fisher Scientific) was used to detect the pERM Abs. To detect F-actin the cells were stained using Alexa Fluor 488 or 647 conjugated to phalloidin (Thermo Fisher). After washing, the cells were resuspended in 250 µl of 1% BSA/PBS and filtered prior to acquisition on the flow cytometer. The flow cytometry gating strategy for bone marrow, spleen, and lung is shown (**Supplementary Figures 1** and **2**) and flow cytometer information is included in the supplement. Because we gated on equal numbers of splenocytes, the increase in G184S BM neutrophils obscures the overall reduction in CD11b expression on both the immature and mature neutrophil subsets. As such we lowered the CD11b expression level used to distinguish the mature and immature bone marrow neutrophils in the G184S mouse bone marrow. Supporting this decision, the immature WT and G184S neutrophils expressed low levels of CXCR2 and Ly6G (data not shown). In contrast, the WT and G184S lung and liver neutrophils exhibited similar levels of CD11b expression.

### BrdU Labeling

BrdU labeling of endogenous neutrophils was modified from previously described (19). Briefly, neutrophil precursors in the mouse BM were labeled *via* intravenous (i.v.) injection of 5-bromo-2′-deoxyuridine (BrdU; 2 mg per mouse; APC (Allophycocyanin) BrdU Flow Kit; BD Biosciences. Two days after BrdU injection, mice received an i.v. injection of 100 µg CD62L antibody or PBS to block transendothelial migration and to reduce neutrophil margination. Blood samples were collected at indicated times and BM, spleen and lung samples were collected at 2 day and 4 day after BrdU injection. The samples were stained for Ly6G, Ly6C, and CD11b, followed by fixation and intracellular labelling of BrdU using an APC-conjugated anti-BrdU antibody as per manufacturer’s instructions (BD Biosciences). The number of cells were counted by a MACSQuant flow cytometer and data acquisition was done on FACSCelesta SORP flow cytometer.

### Neutrophil Mobilization

Neutrophil mobilization to the blood was performed as previously described (20). Mice were injected with CXCL1 (40 μg/kg), AMD 3100 (5mg/kg), KRH 3955 (2.5mg/kg), or PBS only *via* the tail vein. A group of mice received intravenously both AMD3100 and CXCL1. The AMD 3100 was injected 20 min prior to the CXCL1. The Fifty-microliter of whole blood was collected before and 1, 2, and 3 hours after injection from the mandibular plexus or the tail vein. After ACK lysis, the samples were stained with labeled antibodies and counted by a MACSQuant flow cytometer (Miltenyi Biotec).

### Chemotaxis and Migration Assays

Chemotaxis assays were performed using a transwell chamber (Costar) as previously described (21). Where indicated the murine microvascular SVEC4-10EE2 endothelial cell line cells were cultured on the transwell insert until 90% confluence prior to use in the assay. The numbers of cells that migrated to the lower well after a 1 h incubation were counted using a MACSQuant flow cytometer (Miltenyi Biotec). The percent migration was calculated by the numbers of cells of a given subset that migrated into the bottom chamber divided by the total number of cells of that subset in the starting cell suspension and multiplying the results by 100.

### Intracellular Calcium Measurement

Cells were seeded at 10^5^ cells per 100 µl loading medium (RPMI 1640, 10% FCS) into poly-D-lysine coated 96-well black wall, clear-bottom microtiter plates (Nalgene Nunc). An equal volume of assay loading buffer (FLIPR Calcium 4 assay kit, Molecular Devices) in Hank’s balanced salt solution supplemented with 20 mM HEPES and 2 mM probenecid was added. Cells were incubated for 1 hr. at 37°C before adding the indicated concentration of chemokine, fMLP or C5a and then the calcium flux was measured using a FlexStation 3 (Molecular Devices). The data was analyzed with SOFT max Pro 5.2 (Molecular Devices). Data is shown as fluorescent counts.

### Receptor Internalization

BM cells or purified blood neutrophils were rested in RPMI 1640/10 mM HEPES for 30 min at 37°C/5% CO_2_ before immunostaining for Ly6G, Ly6C, and CD11b. Subsequently the cells were stimulated with fMLP (1 µM), solvent (DMSO), CXCL1 (100 ng/ml), or CXCL2 (100 ng/ml), while being maintained at 37°C/5% CO_2_. Thirty minutes later, the cells were added cold 4% PFA (paraformaldehyde) buffer and fixed on ice 30 mins. Cells were stained with CXCR2-PE and analyzed by flow cytometry for cell surface CXCR2 expression.

### Adhesion Assay

Glass-bottom culture dishes (MatTek) were coated with 1 μg/ml ICAM-1 in phosphate-buffered saline (PBS) containing 2 mM MgCl_2_ and 1 mM CaCl_2_ (200 μl/well) at 4°C overnight. The dishes were washed three times with PBS before use. Neutrophils were isolated from BM cells by positive selection using the neutrophil isolation kit (Miltenyi Biotec, Auburn, CA). WT and G184S KI neutrophils were stained by Alexa Fluor 568- and Alexa Fluor 488-conjugated anti-mouse Gr-1 antibodies, respectively. Cells were rested at 37°C for 30 min. Equal numbers of WT and G184S KI neutrophils (1 x 10^5^ cells/100 ul) were transferred to a single tube to which was added 100 ng/ml CXCL2 and 5 mM MgCl_2_. Cells were immediately seeded on a ICAM-1 coated dish and placed in an incubator at 37°C and 5% CO_2_ for 30 min. Images were acquired on a PerkinElmer UltraVIEW spinning disc confocal system (PerkinElmer Life Science, Waltham, MA), with a Zeiss Axiovert 200 inverted microscope (Carl Zeiss Microimaging, Thornwood, NY) equipped with a 40X oil-immersion objective (N.A. 1.3). Images were acquired and the data were processed using ImageJ software (National Institutes of Health, Bethesda, MD).

### Analysis of Neutrophil Aging in Blood

Analysis of neutrophil aging was performed as previously described (3). Briefly, we compared blood drawn at ZT5 or ZT13 in mice housed under a 12h:12h light:dark cycles. We collected 50ul of whole blood at the indicated time points and analyzed blood neutrophil numbers and their expression levels of CXCR4, CXCR2, or CD62L. Blood neutrophils at ZT5 were also analyzed for F-actin levels and pERM levels by intracellular flow cytometry as describe above. Forward light scatter of ZT5 and ZT13 neutrophils was collected by flow cytometry. Previously published RNA-sequencing data (Genome Expression Omnibus accession number GSE86619) was used to determine RGS protein and Gα protein mRNA expression in ZT5 and ZT13 mouse neutrophils (3). After ACK lysis, the samples were stained with labeled antibodies and counted by a MACSQuant flow cytometer (Miltenyi Biotec). After counted cells were stained with isotype controls, CXCR4, CXCR2, or CD62L antibody and acquired by a FACSCanto II or FACSCelesta SORP (BD) flow cytometer and analyzed with FlowJo software (Treestar).

### Neutrophil NETosis

BM Neutrophils (> 95% purity) were resuspended in RPMI medium containing 10% FCS (1 x 10^6^/ml) and incubated for 37°C and 5% CO_2_ for 30 min. To induce NETosis, the cells were exposed to the activating agent PMA (10, 30 nM; Sigma-Aldrich) or fMLP (100, 1000 nM; Sigma-Aldrich) for 0-4 hrs at 37°C. NET induction was terminated by 4% PFA for 15 min. Helix NP™ NIR (0.1 µM; Biolegend) and DAPI (0.3 nM; Biolegend) were added to detect NETs. Data acquisition (Helix NP™ NIR^+^ DAPI^+^ Cells) was done on FACSCelesta SORP (BD) flow cytometer and analyzed with FlowJo software (Treestar).

### Spleen, BM, and Lung Imaging

Immunohistochemistry was performed as previously described (22). For imaging neutrophil recruitments from the BM, mice were injected i.v. with CXCL1 (40 μg/kg) or with an intraperitoneal (i.p.) injection of AMD3100 (5 mg/kg). BM surgery preparation for intravital microscopy was modified from a previously described protocol (23). Blood vessels were outlined by the i.v. injection of 1% EB solution in PBS (Evans Blue Dye, Sigma-Aldrich) at 1 ml/kg. A single incision, starting between the ears and following the head midline until 3-4 mm from the nose area was made with sharp scissors, then skin flaps were separated by pulling toward the sides with forceps. After surgery, the mice were transferred and stabilized onto an imaging stage using an upright setup and a custom-made stage with a head holder (NIH Division of Scientific Equipment and Instrumentation Services). 2-photon laser scanning microscopy was performed with a LEICA SP5 inverted 5-channel confocal microscope (Leica Microsystems) equipped with a 25X water-immersion objective, N.A. 0.7. 2-photon excitation was provided by a Mai Tai Ti : Sapphire laser (Spectra Physics) with a 10 W pump, tuned wavelength rages from 820 – 920 nm. Confocal microscopy of live lung sections was performed as follows. After euthanasia, mouse lungs were inflated with 1.5% of low-melt agarose in RPMI at 37 C. Inflated tissues were kept on ice, in 1% FCS in PBS, and sliced into 300-350 µm sections using Leica VT1000 S Vibrating Blade Microtome (Leica Microsystems). Tissue sections were stained with fluorescently labeled anti-CD31, anti-CD45, and anti-Ly6G antibodies (eBioscience). After staining sections were washed 3 times and cultured in Phenol Red-free RPMI supplemented with 10% FCS, 25 mM HEPES, 50 µM β-ME, 1% Pen/Strep/L-Glu and 1% Sodium Pyruvate). Tissues were allowed to recover for 12 h prior to imaging. Sections were imaged using Leica DMi8 inverted 5 channel confocal microscope equipped with an Environmental Chamber. Diode laser for 405 nm excitation; Argon laser for 488 and 514 nm excitation, DPSS laser for 561; and HeNe lasers for 594 and 633 nm excitation wavelengths were tuned to minimal power (between 1 and 5%). Z stacks of images were collected (10 – 50 µm). Mosaic images of lung sections were generated by acquiring multiple Z stacks using motorized stage to cover the whole section area and assembled into a tiled image using LAS X (Leica Microsystems) software. For time-lapse analysis of cell migration, tiled Z-stacks were collected over time (1 to 4 h).

### Cremaster Muscle, Inguinal Lymph Node, and Liver Preparations for Neutrophil Imaging

For imaging neutrophils in the cremaster muscle, an intrascrotal injection of IL-1β (50 ng in 300 µl saline; R&D Systems) and in some instances an i.p. injection of AMD3100 (5 mg/kg in 200 µl saline; R&D Systems) were used to stimulate acute inflammation in the cremaster muscle and to mobilize neutrophils. Separate saline injections were used as controls. After 90 min, the mice received injections of Avertin (300 mg/kg, i.p.) and fluorescently labeled antibodies (i.v.) to visualize neutrophils and blood vessel endothelium. The isolated cremaster tissue was exteriorized and stabilized onto the imaging stage/insert with the tissue directly contacting the cover glass. The exposed tissue was kept moist with pre-warmed saline (37°C). For imaging neutrophils in lymph nodes, mice received injections of Avertin (300 mg/kg, i.p.) and fluorescently labeled antibodies (i.v.) to delineate neutrophils and venule endothelium. Inguinal lymph nodes were prepared for intravital microscopy as described (24). To image liver neutrophils, the mice received an i.v. injection of saline or ConA (2.5 mg/kg) to induce liver inflammation. Three hours later, the mice received Avertin (300 mg/kg, i.p.) and fluorescently labeled antibodies (i.v.) to outline liver sinusoids, neutrophils, and platelets. To expose the liver an incision was made along the midline on the abdomen. The skin on the left and right side from abdominal muscles were separated, then cut and removed. A second incision along the midline of the abdomen exposed the xiphoid cartilage, and the abdominal muscles were removed by cauterization. An exposed liver lobe was flipped onto the cover glass and covered with a saline-moistened gauze.

### Imaging Neutrophils

Once stabilized onto an imaging stage/insert the mouse received isoflurane (Baxter; 2% for induction of anesthesia, and 1 – 1.5% for maintenance, vaporized in an 80:20 mixture of oxygen and air), and placed into a temperature-controlled chamber. A Leica SP8 inverted 5 channel confocal microscope equipped with a motorized stage, 4 hybrid ultra-sensitive detectors, and argon and helium neon lasers, and a 25x water-immersion objective (1.0 N.A) was used for imaging (Leica Microsystems). For cremaster imaging, sequential scans of the 488-nm channels for anti-Gr-1 AlexaFluor-488 labeled neutrophils (10 µg/mouse; clone RB6-8C5) and 633-nm channels for anti-CD31-AlexaFluor-647 labelled unbranched 25-40 µm venules (10 µg/mouse; clone 390) were acquired. For the inguinal lymph nodes, sequential scans of the 488-nm channels for anti-Gr1 AlexaFluor-488 labeled neutrophils (10 µg/mouse) and 561-nm channels for anti-CD31-AlexaFluor-555 labeled HEVs (10 µg/mouse) were captured following laser damage. For the liver, scans of the 488-nm channels were used to detect anti-Gr1 AlexaFluor-488 labeled neutrophils (10 µg/mouse), 561-nm channels for anti-CD31-AlexaFluor-568 labeled sinusoids (10 µg/mouse), and 633-nm channels for anti-GPIbβ-DyLight649 labelled platelets (2µg/mouse) (25). Images were acquired at a resolution of 1024 x 1024 pixels, which corresponds to a voxel size of approximately 0.25 x 0.25 x 0.7 µm in the x, y, z planes. Image stacks of optical sections ~ 1 µm thickness were routinely acquired at 20 – 40 sec intervals, and with the incorporated resonance scanner of 8,000 Hz. This methodology allowed acquisition of full 3D confocal images of cremaster venules, HEVs/laser damage, and liver sinusoid, which yielded high-resolution 4D videos (real-time in 3D) of dynamic events.

### Image Processing and Analysis of Leukocyte Dynamics

After acquisition, sequences of z-stack images were analyzed with Imaris Software (version 9.5.0, Bitplane AG, Zurich, Switzerland). For cremaster confocal intravital imaging sequential z-sections of stained cells in time frames were acquired for 3D reconstruction and surface modeling of representative. The 3D cell surfaces were then tracked using tracking algorithm of Imaris. The tracked cells were then divided into two groups (attached to the vasculature and free) utilizing the filtering function of Imaris and mean intensity threshold of the channel representing veins.

### Histopathology

Mice were injected with ConA (2.5 mg/kg in in 90 µl saline, i.v.) for liver confocal intravital imaging experiments. Mice were sacrificed after ~2 – 4 hours of imaging. Livers and Lungs were collected and fixed in 10% formalin for 7 days. Samples were embedded in paraffin, cut into 5 µm sections, stained for hematoxylin and eosin (H&E), Histoserv, Inc. The pathology slides were reviewed by Dr. Victoria Hoffmann, Division of Veterinary Resources, Office of the Direction, National Institutes of Health.

### Quantification and Statistical Analysis

*In vivo* results represent samples from three to six mice per experimental group. Results represent mean values of at least triplicate samples for ex vivo experiments. Data analysis was performed using Graph-pad Prism 8 (La Jolla, CA, USA) with *t* test or ANOVA. Results are presented as mean ± standard error of mean (S.E.M.). Statistical significance was assessed by p < 0.05 to be considered statistically significant. *p-*values for each comparison are indicated in the figure legends.

## Results

### Neutrophil Overload in G184S BM Reconstituted Mice Despite Neutropenia

**Figure 1:**
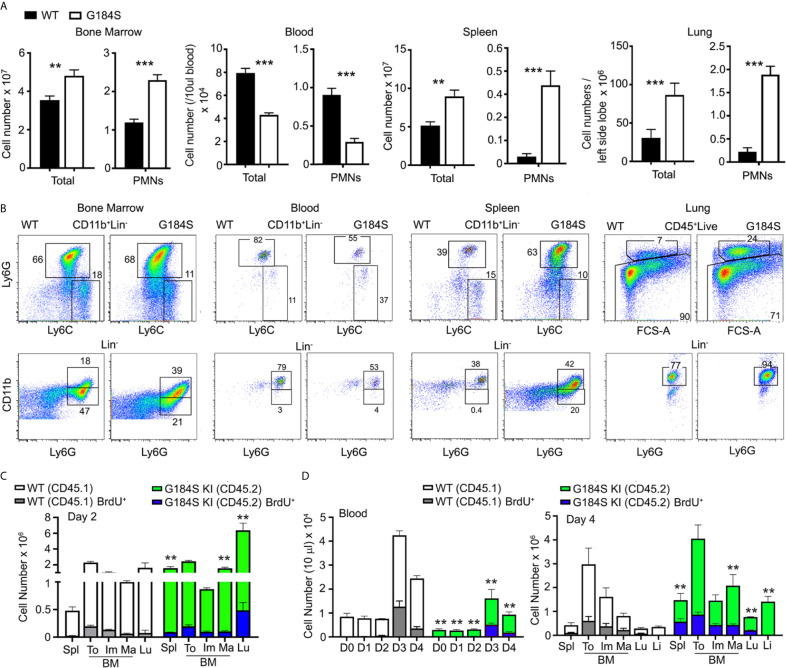
Neutrophil overload despite neutropenia in G184S BM reconstituted mice. **(A)** A comparison of total leukocyte and neutrophil (PMN) blood cell counts between WT and G184S BM reconstituted mice in a single tibia BM, 50 µl of blood, one spleen, and both lungs. Neutrophils are identified as CD11b^+^/Ly6C^inter^/Ly6G^+^ cells by flow cytometry. **(B)** Representative flow cytometry patterns and numbers of leukocytes and neutrophils in BM, blood, spleen, and lung. **(C)** Cell count comparison of WT *vs* G184S neutrophil BrdU positivity and distribution 2 days after BrdU injection in 1:1 (WT:G184S) mixed chimeras. Spl, spleen; To, total neutrophils; Im, immature; Ma, mature; Lu, lung. **(D)** Cell count comparison of WT *vs* G184S neutrophil BrdU positivity and distribution 2 days after BrdU injection and CD62L antibody treatment in mixed chimeras. Graph on the left shows the neutrophil count in blood from days 0 to 4 (D0 – D4), with BrdU and CD62L antibody administered on D2. Graph on the right shows the WT *vs* G184S BrdU positivity and neutrophil distribution on D4. Statistics: data are means ± SEMs, and analyzed using unpaired Student’s *t*-test comparing G184S with WT. Results are from 3 separate experiments done in duplicates from 3 mice. *p < 0.05, **p < 0.005 and ***p < 0.0005.

We determined the numbers of neutrophils in the blood, BM, and spleen of mice reconstituted with WT or G184S BM. Neutrophils lack the lineage markers B220, CD3, CD4, CD8, CD11c, NK1.1, c-kit, and TCR*γ*δ; but express CD11b and Ly6G. We assessed neutrophil maturity by the level of CD11b expression on Ly6G^+^ cells (6, 26). Because we gated on equal numbers of splenocytes it obscures the reduction in the reduction in CD11b on both the immature and mature G184S neutrophils. Representative flow cytometry patterns and the numbers of leukocytes and neutrophils at the different sites are shown. The G184S BM reconstituted mice have a reduced number of blood neutrophils with expanded populations in the spleen, lung, and the BM (**Figure 1A**). The spleen had a 10-fold, the lung an 8-fold, and the BM a 2-fold expansion of neutrophils compared to WT while the blood had a 3.5-fold reduction. The G184S BM reconstituted mice also had a relative increase in mature neutrophils in the BM (CD11b^high^ and Ly6G^+^) compared to WT BM reconstituted mice, while they had an increase of immature cells (CD11b^low^Ly6G^+^) in the spleen (**Figure 1B**).

The neutropenia in the setting of an expanded BM population of mature neutrophils suggests a BM egress defect. Yet a BM egress defect cannot explain the peripheral neutrophil expansion noted in these mice suggesting a more complex phenotype. To assess whether the concomitant presence of WT neutrophils would normalize the G184S neutrophil distribution we made mixed BM chimeric mice. C57Bl/6 CD45.2 mice were reconstituted with 50% CD45.2 WT BM and 50% CD45.1 G184S BM. After 6-8 weeks we assessed the number of neutrophils in various locations using CD45.1 and CD45.2 monoclonal antibodies to distinguish the two genotypes. We performed two sets of experiments, in the first we injected the mixed chimeric mice with BrdU two days prior to sacrificing the mice for analysis (**Figure 1C**). In the second we again injected BrdU, but on day 2 we treated the mice with a CD62L antibody to reduce neutrophil transendothelial migration; and sacrificed the mice on day 4 (**Figure 1D**). In sum, the mixed chimera BM contained a nearly equal ratio of WT and G184S neutrophils, although the G184S cells predominated among the mature BM neutrophils. The WT neutrophils exceeded the G184S cells in the blood, 70% versus 30%, while the lung, liver, and spleen each contained a 3 to 4-fold excess of G184S versus WT neutrophils. The CD62L treatment raised the blood neutrophil counts causing a 5 to 6-fold increase compared to basal for both the WT and G184S cells. The BrdU labeling suggested that the neutrophil production rates for the two genotypes did not differ appreciably (**Figure 1D**). The coexistence of the WT and G184S neutrophils in the mixed chimeric mice partially corrected the BM expansion, failed to correct the neutropenia, and partially corrected the peripheral neutrophil overload noted in the G184S straight chimeric mice. These results suggest that the neutropenia in the G184S mice results not only from an egress defect, but also from a short intravascular half-life. The loss of RGS protein/Gα_i2_ interactions leads to expanded pools of neutrophils in spleen, lungs, and the liver.

**Figure 2:**
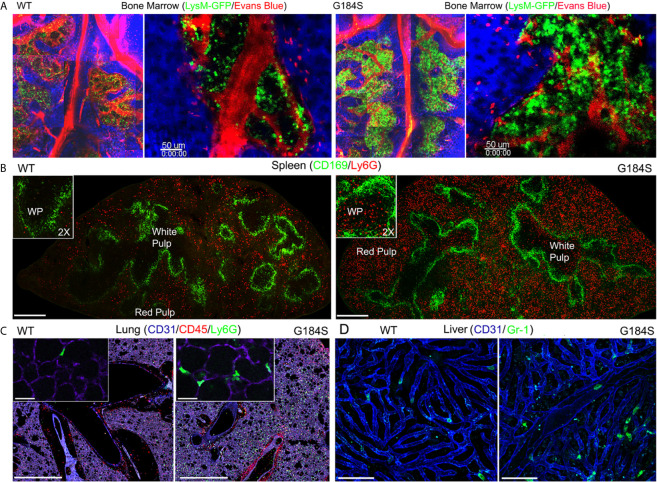
Distribution of WT and G184S neutrophils. **(A)** Two-photon (2P) microscopy images of skull BM in WT *vs* G184S mice with LysM-GFP neutrophils (green) and Evans Blue labeled blood vessels (red), and collagen/tissue detected by second harmonic generation signal (blue). Right panels are stitched 2P images spanning the BM niches. Right panels show BM niches in higher magnification. Scale bars = 50 µm. **(B)** Confocal microscopy images of WT (left panel) *vs* G184S (right panel) spleens from BM reconstituted mice. CD169 immunostaining (green) separates areas of white pulp and red pulp with Ly6G stained neutrophils (red). Scale bar = 400 µm. The insert images show a higher magnification (2X) focusing on the white pulp area. **(C)** Confocal live lung section images of WT (left panel) *vs* G184S (right panel) BM reconstituted mice. Pulmonary vasculatures (purple) identified with CD31 immunostaining, CD45 and Ly6G immunostaining for leukocytes (red) and neutrophils (green), respectively. Scale bars = 1000 µm. The insert images show a higher magnification focusing on the alveoli. Scale bars = 30 µm. **(D)** Confocal intravital microscopy (IVM) liver images of WT (left panel) *vs* G184S (right panel) BM reconstituted mice. The liver sinusoids (blue) are outlined by CD31 immunostaining, while the neutrophils (green) are identified by Gr-1 staining. Scale bars = 60 µm. Results are representative images from 3-5 mice for each study.

To address the localization of the G184S neutrophils in the BM, spleen, lung, and liver we used a combination of immunohistochemistry and intravital microscopy. For these experiments, we relied on G184S and WT BM reconstituted mice, or occasionally on non-reconstituted WT and G184S mice. Of note the spleens isolated from the G184S mice and the G184S BM reconstituted mice were nearly twice the weight of those isolated from the WT and WT BM reconstituted mice (data not shown). Two-photon microscopy of the skull BM confirmed the expanded neutrophil BM niche in the G184S mice. Mice with the LysM-GFP transgene crossed onto a G184S background were used for the BM imaging. We visualized the BM vasculature by infusing Evans blue dye into the bloodstream. Stitched sets of 2-photon images spanning the BM niches are shown. Higher magnification images show the high neutrophil density in the neutrophil niches of the G184S BM (**Figure 2A**). The spleens from the G184S mice Next, we used confocal microscopy to examine fixed spleens from mice reconstituted with WT or G184S BM and immunostained with CD169 to delineate the splenic marginal zone and the separation between the white pulp and red pulp; and Ly-6G to identify neutrophils. Stitched confocal images show the larger spleen size and the markedly expanded neutrophil population in the splenic red pulp of G184S BM reconstituted mice (**Figure 2B**). Higher magnification inserts demonstrate the infiltration of G184S neutrophils into the white pulp. Normally, in young mice neutrophils do not enter the white pulp or lymph node follicles (27, 28). As mice age, rare neutrophils can be found in the white pulp, and infection or LPS challenge can also cause neutrophils to enter the white pulp or lymph node follicles (27, 29). Live confocal microscopy of lung sections immunostained with CD31 and Gr-1 antibodies confirmed the marked neutrophil expansion in the lungs of the G184S BM reconstituted mice (**Figure 2C**). CD31 immunostaining delineated the pulmonary endothelial cells and outlined the pulmonary vasculature. Higher magnification inserts show that the G184S neutrophils largely resided within the pulmonary vasculature. Finally, we used intravital confocal microscopy to image the liver outlining the liver sinusoids by injection of labeled CD31 and the neutrophils by injecting labeled Gr-1 antibody. At baseline we found relatively few neutrophils in the superficial sinusoids of the WT BM reconstituted mice livers while we consistently observed neutrophils in the liver sinusoids of the G184S reconstituted mice (**Figure 2D**). These data confirm the expanded BM niche and demonstrates that the G184S neutrophils accumulate in the splenic red pulp, lung vasculature, and liver sinusoids. These results suggest that the loss of Gα_i2_/RGS protein interactions in neutrophils dramatically expands the marginated pool of neutrophils.

### Together CXCR2 Agonism and CXCR4 Antagonism Overcomes the Neutrophil Mobilization Defect in the G184S Mice

**Figure 3:**
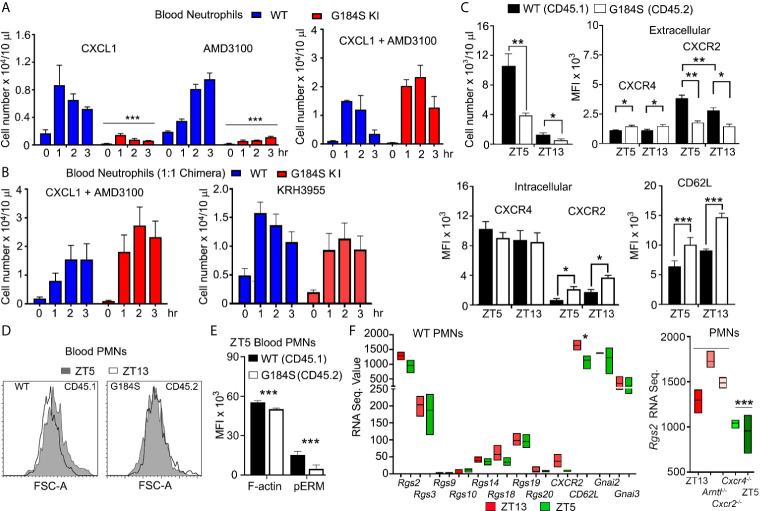
Poor neutrophil mobilization and disrupted neutrophil aging in G184S BM reconstituted mice. **(A)** Left graph, circulating blood neutrophil counts in WT *vs* G184S BM reconstituted mice after injections of CXCL1 (i.v.) or AMD3100 (i.p.). Blood neutrophil counts were monitored from 0 – 3 hours after CXCL1 or AMD3100 injections. Right graph, levels of circulating blood neutrophils in WT *vs* G184S BM reconstituted mice after injections of CXCL1 (i.v.) and AMD3100 (i.p.) 20 min apart. Blood neutrophil counts were monitored from 0 – 3 hours after CXCL1 + AMD3100 injections. **(B)** Circulating blood neutrophil counts in WT *vs* G184S 1:1 BM reconstituted mice after injection of either CXCL1 with AMD3100 (left) or KRH3955 alone (right). Blood neutrophil counts were monitored from 0 – 3 hrs after each treatment. **(C)** Top left graph, circulating WT *vs* G184S blood neutrophil count at ZT5 and ZT13. Top right graph, flow cytometry analysis of surface CXCR4 and CXCR2 expressions of WT *vs* G184S blood neutrophils at ZT5 (aged) and ZT13 (fresh). Bottom left graph, analysis of intracellular CXCR4 and CXCR2 levels. Data are expressed as mean fluorescence intensities (MFI). Bottom right graph, MFI data (left graph) obtained from flow cytometry analysis of surface CD62L levels of WT *vs* G184S blood neutrophils at ZT5 and ZT13. **(D)** Representative flow cytometry result comparing WT and G184S blood neutrophil forward light scatter. Blood neutrophils collected from 1:1 chimeric mice at ZT5 or ZT13 analyzed for forward light scatter. **(E)** Flow cytometry analysis of cortical actin and pEzrin levels of WT *vs* G184S blood neutrophils at ZT5. **(F)** Relative mRNA expression data obtained from RNA seq experiments. RNA sequence value (arbitrary unit) comparing RGS proteins, *Cxcr2*, *CD62L*, *Gnai2*, and *Gnai3* mRNA expression levels of WT blood neutrophils at both ZT5 and ZT13. Right graph shows *Rgs2* mRNA sequence data from neutrophils isolated from WT mice and from neutrophils lacking indicated genes. Statistics: data are means ± SEMs analyzed using unpaired Student’s *t*-test or 1-way ANOVA. *p < 0.05, **p < 0.005 and ***p < 0.0005.

We had previously found that peritoneal inflammation did not efficiently recruit G184S neutrophils into the bloodstream (17). To better understand the mobilization defect we first tested whether raising CXCL1 blood levels to trigger a strong CXCR2 recruitment signal (30), would mobilize the G184S BM neutrophils (**Figure 3A**). Injection of WT mice increased blood neutrophils 8-fold, while blood G184S neutrophils increased, they did not reach the basal level found in WT mice. Next, we tried the CXCR4 antagonist AMD3100, which increases blood neutrophil numbers perhaps by reversing the BM CXCL12 gradient (31, 32). Again, while AMD3100 treatment increased the blood WT neutrophils, the blood neutrophil numbers in G184S mice did not reach the basal level found in WT mice (**Figure 3A**). We also assessed the impact of CXCL1 and AMD3100 administration on the BM niche using intravital imaging. Initial comparison one hour after PBS injection revealed the increased density of LysM-GFP expressing cells in the G184S BM as noted previously, (**Supplementary Figure 3** and **Supplementary Video 1**). We also noted an increased motility of the bone marrow G184S neutrophils. Most of the WT neutrophils oscillated in place, while many of the G184S neutrophils actively moved within the bone marrow niche, often disappearing from the imaging space when tracked over 10 minutes. The tracking data documented their increased velocity and greater displacement. In both sets of mice, we observed relatively few LysM-GFP positive cells in the BM vasculature. The administration of CXCL1 or AMD3100 increase the number of neutrophils in the vasculature, more evident with CXCL1 than AMD3100, and in the WT versus the G184S mice. Both AMD3100 and CXCL1 increased the motility of WT BM neutrophils without obviously affecting the enhanced motility of the G184S neutrophils (**Supplementary Figure 3** and **Supplementary Video 2**). Together these results reveal an increase in the basal motility of G184S neutrophils along with an underlying defect in their ability to be mobilized to the blood by raising CXCR2 signaling or by reversing the CXCL12 gradient.

To provide a stronger mobilization signal, we co-administered AMD3100 and CXCL1. When given simultaneously both the WT and G184S mice rapidly died, however when staggered, AMD3100 followed 20 minutes later by CXCL1, the mice suffered no apparent ill effects. The staggered agents raised the blood level of WT neutrophils approximately 14-fold, nearly two-fold higher than either alone. The G184S neutrophils increased 40-fold above the basal exceeding the number of neutrophils mobilized in WT mice (**Figure 3A**). Intravital microscopy of BM revealed an increase in intravascular neutrophils in both the WT and G184S BMs (**Supplementary Figure 3**). We repeated the co-administration experiment using the 1:1 mixed BM chimeric mice. Treatment of with AMD3100 and CXCL1 led to a 10-fold increase and 40-fold increase in the WT and G184S blood neutrophils, respectively (**Figure 3B**). We also tested a second CXCR4 antagonist, which does not reverse the CXCL12 BM gradient, but acts as a true CXCR4 antagonist (31). Surprisingly, KRH3955 efficiently mobilized both WT and G184S neutrophils without having to co-administer CXCL1 (**Figure 3B**). These data indicate the loss of Gα_i2_/RGS protein interactions cause a misbalance between the CXCR4-mediated retention signal and the CXCR2-mediated recruitment in the BM. They also show that normal neutrophil BM egress depends upon RGS proteins limiting a Gα_i2_ mediated retention signal.

### Neutrophil Aging in G184S Mice

Once neutrophils are released from the BM, neutrophil aging begins. Over time neutrophil CXCR4 levels rise; CXCR2 and CD62L levels decline; cortical actin decreases; and microvilli collapse. Aged and “fresh” neutrophils predominate in the blood at Zeitgeber time 5 (ZT5, 5 hours after lights are on) and ZT13, respectively (26). These changes affect neutrophil BM release, neutrophil recruitment to inflammatory sites, and peripheral neutrophil clearance. To determine if the G184S neutrophils aged appropriately, we first assessed the above receptors on neutrophils collected from different sites at ZT5 using the 1:1 chimeric mice. The G184S BM neutrophils had higher CXCR4 membrane expression, more evident on the mature BM G184S neutrophils. The G184S BM neutrophils also had lower CXCR2 levels, which was accompanied by increased intracellular CXCR2 (**Supplementary Table 1**). The G184S lung, liver, and splenic neutrophils all had significantly reduced levels of CXCR2 and CD62L; but their CXCR4 expression varied, slightly depressed on G184S lung neutrophils, and slightly elevated on liver and splenic neutrophils (**Supplementary Table 2**). Overall, the residential G184S neutrophils had slightly higher membrane CXCR4 levels, and lower amounts of CXCR2 and CD62L finding suggestive of a more aged phenotype.

Next, we checked for appropriate age-related changes by collecting blood at ZT5 and ZT13 for analysis. The G184S neutropenia was present at both ZT5 and ZT13. At each time point the G184S blood neutrophils had higher CXCR4 and lower CXCR2 extracellular expression levels compared to WT neutrophils (**Figure 3C**). Surprisingly, we noted that in the mixed chimera mice, the WT neutrophils had a slightly higher extracellular CXCR2 expression level at ZT5 than at ZT13, opposite of the reported expression variation (3). Total WT blood neutrophil CXCR2 expression (intracellular plus extracellular) did not differ between the time points, while the G184S neutrophils had a higher total CXCR2 expression level at ZT13 compared to ZT5. Compared to the WT cells ZT5 G184S neutrophils had elevated CD62L expression, and the ZT13 G184S neutrophils had even higher levels. However, we noted a similar % decline in CXCR2 expression between ZT13 and ZT5 in both sets of neutrophils (**Figure 3C**). The shape changes that normally accompanies neutrophil aging did not occur in the G184S neutrophils (**Figure 3D**) and the ZT5 G184S neutrophils had reduced amounts of cortical actin and low pERM levels (**Figure 3E**), consistent with microvilli loss (3). Dephosphorylation of ERM proteins (ezrin, radixin, and moesin) is accompanied by a collapse of microvilli and can be triggered by chemokine receptor signaling. Thus, while G184S blood neutrophil numbers exhibited a normal diurnal variation, their expression pattern of homing receptors, cell shape changes, and cortical actin levels did not conform to the aging patterns observed with the WT neutrophils.

To determine whether changes in RGS protein expression accompany normal neutrophil aging, we checked previously published RNA sequence data (4). We found that “young” neutrophils tended to have higher levels of RGS protein expression, however the differences did not reach statistical significance. However, a comparison of *Rgs2* mRNA expression in young neutrophils (ZT13, Arntl deficient, and Cxcr2 deficient) versus aged neutrophils (ZT5 and CXCR4 deficient) suggested that *Rgs2* expression may be impacted by a diurnally regulated signal (**Figure 3F**). *Gnai2* and *Gnai3* mRNA expression levels remained stable, although ZT13 cells had higher *Gnaq* mRNA levels (223 *vs*. 130, p < 0.02). Together these data indicate that changes in Gα_i_ protein expression is unlikely to impact neutrophil aging, while RGS2 warrants additional study.

### Appropriate Chemoattractant Receptor Downregulation, but Abnormal Basal and Chemoattractant Elicited Responses in G184S Neutrophils

Besides age related changes ligand exposure can impact chemoattractant expression levels. Using the 1:1 mixed chimeric mice we assessed the changes in CXCR4 and CXCR2 expression following *in vivo* administration of KRH3955, and CXCR2 expression following exposure to different ligands *in vitro*. Following KRH3955 administration membrane CXCR2 declined on both the WT and G184S PMNs, although the G184S neutrophil expression declined more gradually (**Supplementary Figure 4A**). Since the WT blood neutrophils had a higher initial expression level, by three hours post KRH3955 no significant difference remained. The blood G184S neutrophil higher membrane CXCR4 levels declined gradually in parallel with the WT neutrophils after KRH3955 injection. Overall, the % decline in CXCR4 and CXCR2 were not significantly different (**Supplementary Figures 4B, C** **)**. *In vitro* exposure to CXCL1, CXCL2, or to the chemoattractant fMLP led to similar declines in the WT and G184S neutrophil CXCR2 expression (**Supplementary Figures 4D, E** **)**. Overall, the loss of Gα_i2_/RGS protein interactions affected both CXCR2 and CXCR4 expression and signaling (see below), which in turn likely impacted neutrophil aging. An alteration in ligand induced downregulation of CXCR2 or CXCR4 in the G184S neutrophils does not explain the noted changes in receptor expression.

**Figure 4:**
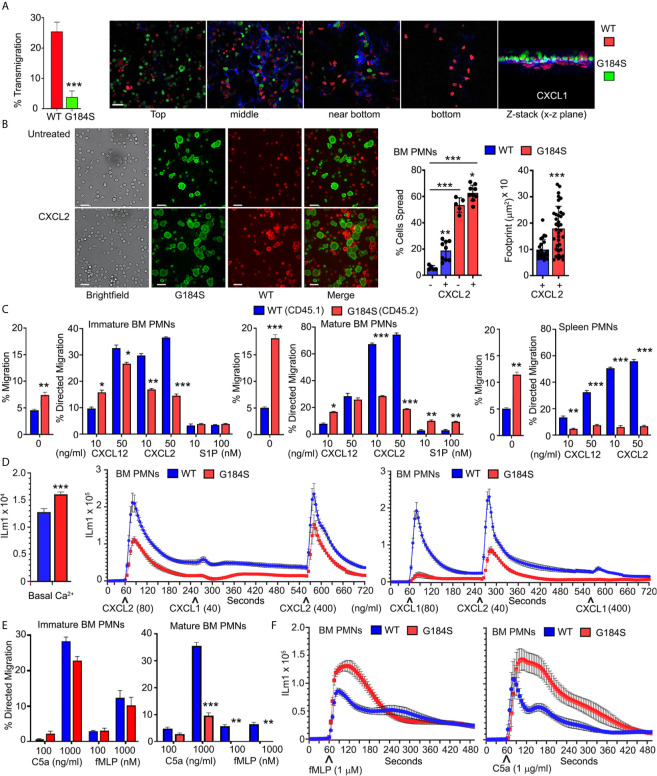
Disrupted basal and chemoattractant elicited responses in the G184S neutrophils. **(A)** Transmigration frequency (%) of WT *vs* G184S BM neutrophils to CXCL1 stimuli *in vitro* across SVEC4-10 coated transwell filters (left graph). Results are from 3 separate experiments done in duplicates. Confocal images (right panels) show the extent of differentially labeled WT (red) *vs* G184S (green) BM neutrophils crossing the endothelial barrier (blue) during the migration assay (top, middle, near bottom, & bottom). The Z-stack image viewed from the x-z plane (far right). Scale bar = 30 µm. **(B)** Representative brightfield and confocal images showing basal and CXCL2 triggered WT and G184S BM neutrophil adhesion *in vitro*. Differentially labeled WT (red) and G184S (green) neutrophils treated with CXCL2 (bottom panels) or untreated (top panels) were seeded onto ICAM-1 coated plates. Scale bars- 40 µm brightfield, 20 µm fluorescent images. Neutrophil % spread and footprint sizes were quantified (right). **(C)** *In vitro* chemotaxis assays assessing the migratory capacities of WT *vs* G184S immature (left graph), mature (middle graph) BM neutrophils, and splenic neutrophils with increasing concentrations of CXCL12, CXCL2 and S1P. Shown are the percentages that specifically migrated, with non-specific migration shown as ‘0’ treatment. **(D)** Intracellular calcium levels. Left graph, WT and G184S BM neutrophil basal fluorescence from 5 matched pairs of mice is shown. Right graph, changes in intracellular calcium levels were monitored over 12 minutes (720 sec) in WT and G184S BM neutrophils exposed to CXCL2 and CXCL1, then re-stimulation with a higher dose of CXCL2. Intracellular calcium response was again monitored over 12 minutes in WT and G184S BM neutrophils exposed to CXCL1, CXCL2, then re-stimulation with a higher dose of CXCL1 (bottom). Results from neutrophils prepared from 2 WT and 2 G184S bone marrow reconstituted mice done in triplicate. **(E)** Chemotaxis assays assessing migratory capacities of WT *vs* G184S immature (left) and mature (right) BM neutrophils with increasing concentrations of C5a or fMLP *in vitro*. **(F)** The intracellular calcium response of WT *vs* G184S BM neutrophils monitored over 8 minutes (480 sec) after stimulations with fMLP (left) or C5a (right). Results from neutrophils prepared from 3 WT and 3 bone marrow reconstituted mice done in duplicate. Statistics: data are means ± SEMs, then analyzed using student t-test or ANOVA. Results are from n ≥ 3 experiments done in duplicate or triplicate. *p < 0.05, **p < 0.005 and ***p < 0.0005.

Neutrophil recruitment from the blood stream to inflamed or injured tissue is a sequential multi-step process, which includes neutrophil tethering, rolling, firm adhesion, exploration, transmigration, and chemotaxis (33). Other than the initial neutrophil tethering and rolling, all these steps depend upon intact Gα_i_ signaling. An *in vitro* model to the cascade of events that follows neutrophil tethering and rolling is to determine whether neutrophils can cross an endothelial cell line coated porous membrane in response to a chemoattractant gradient. Using a transwell filter (3 µm pores) coated with the murine endothelial cells line (SVEC4-10), we compared WT and G184S neutrophils responses to a CXCL1 gradient. While many WT neutrophils crossed the endothelial cell barrier the G184S neutrophil largely failed (**Figure 4A**). We visualized the difficulty G184S PMNs had in crossing by differentially labeling the two sources of neutrophils and imaging across the cell barrier. This showed that the G184S neutrophils had poorly penetrated the barrier appearing to be stuck on the endothelial cells (**Figure 4A**). To check basal and CXCR2 mediated adhesion, we plated differentially labelled WT and G184S neutrophils on ICAM-1 coated plated in the presence or absence of CXCL2. We allowed the cells to adhere for 30 minutes before cell imaging. Surprisingly, in the absence of CXCL2 pre-exposure, we found 11-fold more G184S neutrophils attached and spread on the ICAM-1 coated plates than WT cells (**Figure 4B**). Pre-exposure to CXCL2 prior to plating enhanced 4-fold the number of spread WT neutrophils, however, the percentage spread failed to reach the % G184S neutrophil spread without CXCL2. Furthermore, due to their high background adhesion, CXCL2 minimally affected the % G184S neutrophil that spread. A comparison of footprint sizes of the CXCL2-activated neutrophils revealed a dramatic increase among the G184S neutrophils compared to the WT cells (**Figure 4B**). Thus, the difficulty that G184S neutrophils cells had in crossing the SVEC4-10 coated membrane may be secondary to their augmented adhesiveness.

Reasoning that removal of the endothelial cell line would better allow the G184S neutrophils to migrate into the lower chamber, we repeated the transwell experiments without the SVEC4-10 cells. This revealed that many more G184S BM neutrophils than WT cells could spontaneously migrate into the bottom chamber (**Figure 4C**). Despite this high basal migration, CXCL12 elicited comparable levels of specific neutrophil migration, however, CXCL2 did not. The mature G184S BM neutrophils also migrated to sphingosine-1 phosphate (S1P), even slightly exceeding the WT cell response (**Figure 4C**). Next, we checked the G184S neutrophils that resided in the spleen. Like the BM neutrophils many more splenic G184S neutrophils than WT cells migrated spontaneously, but their specific responses to CXCL2 and CXL12 were much worse (**Figure 4C**). These results indicate that RGS proteins suppress basal adhesiveness and motility. This helps neutrophils to respond appropriately when encountering chemoattractants.

As CXCR2 triggered migration showed the most severe impairment, we tested proximal CXCR2 signaling by measuring the intracellular calcium responses following sequential exposure to CXCL1 and CXCL2, which have been shown to mediate neutrophil TEM *in vivo* (30). At baseline the G184S neutrophils had elevated intracellular calcium levels (**Figure 4D**). Relative to WT neutrophils, both CXCL1 and CXCL2 elicited substandard increases in intracellular calcium in the G184S neutrophils (**Figure 4D**). We also tested the migration and intracellular calcium response to two additional neutrophil chemoattractants, fMLP and C5a. Immature BM neutrophils migrated comparably to both, while mature G184S BM neutrophils again showed significant defects (**Figure 4E**). Surprisingly, while C5a and fMLP elicited suboptimal migration, they triggered enhanced intracellular calcium responses (**Figure 4F**). This argues that the proximal signaling pathway leading to calcium mobilization following Gα_i_ activation is intact in the G184S neutrophils.

### G184S Neutrophils Exhibit Defective *In Vivo* Recruitment and Directed Migration

**Figure 5:**
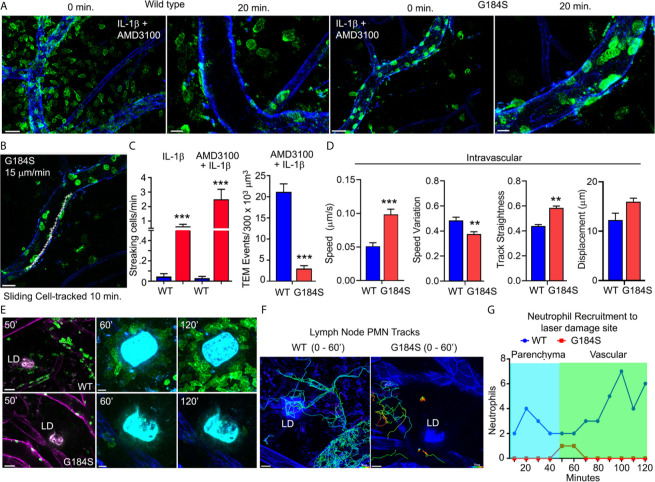
*In vivo* G184S neutrophils transmigrate poorly and show impaired directional migration. **(A)** Representative confocal IVM images showing *in vivo* WT *vs* G184S neutrophil migration in 3 hrs IL-1β (i.s.) and AMD3100 (i.p.) treated cremaster muscle. Shown are images captured at 0 minutes and 20 min after the imaging commenced. 0 min, scale bar = 30 µm; 20 min, scale bar = 10 µm. **(B)** A representative confocal IVM image shows tracking (white +) of an intravascular G184S neutrophil crawling/sliding along the vessel lumen over 10 minutes. Scale bar = 20 µm. **(C)** Quantification of streaking and transmigrated cells over a 250 µm vessel segment in WT *vs* G184S mice treated with either IL-1β alone or IL-1β and AMD3100. **(D)** Transmigrated cells inside the vessel segment in WT *vs* G184S mice treated with IL-1β and AMD3100. Speed, speed variation, track straightness, and displacement from tracking intravascular WT *vs* G184S neutrophils in mice treated with IL-1β and AMD3100 over 30 minutes. **(C, D)** Results are from mice following 90 min treatment of IL-1β and AMD3100. **(E)** Representative confocal IVM images of *in vivo* WT (top panels) *vs* G184S (bottom panels) neutrophils from BM reconstituted mice in inguinal lymph node (LN) at various time points after 2P laser damage. Images of Gr-1 immunostained neutrophils (green) migrating within the LN after laser damage. LN vasculatures (magenta) are labeled by CD31 immunostaining (left panels). Scale bars = 30 (left panels), 10 µm (middle, right panels). **(F)** Representative confocal IVM images showing tracking of WT (left) and G184S (right) neutrophils around the LD site for 60 minutes after LD. Scale bar = 20 µm. **(G)** Quantification of accumulating WT *vs* G184S neutrophils around the LD site from 0 – 120 minutes after LD damage. Migrating cells are divided into either those originating from the LN parenchyma or from blood vessels. All results are representative from imaging 3-5 WT and 3-5 G184S BM reconstituted mouse. **p < 0.005 and ***p < 0.0005.

To visualize how these *in vitro* defects translated *in vivo* we used the intravital microscopy to assess neutrophil behavior in the cremaster muscle blood vessels of a mouse (34). We used IL-1β to recruit neutrophils and injected fluorescent CD31 and Gr-1 antibodies to outline the vasculature and neutrophils, respectively. Because of the poor BM release of G184S neutrophils into the bloodstream, we also injected AMD3100 intraperitoneally. The addition of AMD3100 released more WT and G184S neutrophils into the circulation. This led to a marked increase in WT neutrophils in the cremaster blood vessels and massive amounts of transmigration **(** **Figure 5A** and **Supplementary Video 3**). While many G184S neutrophils arrived, relatively few transmigrated into the interstitium. The massive TEM observed in WT mice was mostly due to efficient crossing of WT neutrophils through the endothelium after initial adherence, and their effective ability to find transmigration sites (**Supplementary Video 4**). In contrast, many G184S neutrophils engaged the endothelium, but failed to remain firmly attached, flying away, or sliding along the endothelium. The few G184S neutrophils that underwent TEM had either spent significant longer time breaching the endothelial barrier or showed extensive intraluminal crawling before TEM (**Supplementary Video 5**). We tracked 18 G184S neutrophils in a vessel segment over a 20-minute period after 90 minutes of IL-1β and AMD3100 treatment. Five minutes after the initial image, 12 cells had departed the imaging field. Over the ensuing 15 minutes, 1 more left and 2 transmigrated. One cell slid along the endothelium for 10 minutes before departing (data not shown, **Figure 5B**). A comparison of the WT and G184S neutrophils shows many streaking (non-adherent or rolling cells) G184S cells compared to WT neutrophils and fewer transmigration events both with IL-1β alone and with the addition of AMD3100 (**Figure 5C**). To evaluate neutrophil dynamics, we divided the tracked cells into two groups (attached to the vasculature and free, **Supplementary Figure 5**). A comparison of tracked cells within the blood vessels (attached cells) showed an increased speed and straightness of the G184S PMN tracks (**Figure 5D**). We also tracked neutrophils outside of the cremaster blood vessels and migrating within the interstitium (free cells). Representative tracks are shown for both WT and G184S interstitial neutrophils. Once the G184S PMNs transmigrated, they moved faster resulting in longer tracks and greater displacement (**Supplementary Figures 6A, B** **)**. Visualizing individual cells showed that the WT cells tended to adopt a polarized morphology with Gr-1 immunostaining concentrated on the uropod. In contrast, many of the G184S PMNs failed to properly polarize often exhibiting multiple lamellipodia and no clear uropod. Snapshots of representative cells are shown. Analyzing the movements of individual cells revealed the distorted movement of the leading and trailing edges (**Supplementary Figures 6C, D** **)**. Thus, the G184S neutrophils are poorly recruited into inflammatory sites often failing to firmly adhere to the endothelium and undergo transmigration. Those few cells that transmigrate move quickly, but erratically.

To test directed migration *in vivo*, we examined the recruitment of G184S neutrophils to a site of sterile injury. Recruitment to a sterile injury in the skin involves CXCR2, FPR2, and LTB4R1 (35). It occurs in three phases; an initial Gα_i_-dependent scouting phase, a secondary amplification phase, and a stabilization phase (36). Using BM reconstituted mice, we labeled the vasculature and neutrophils with intravenously injected fluorescent CD31 and Gr-1 antibodies, respectively. Since the Gr-1 antibody labeled the intravascular neutrophils better than the lymph node resident neutrophils, we could distinguish the two-populations based on their fluorescence intensity. Within 5-10 minutes of the laser damage (LD) WT resident neutrophils appeared in the imaging field as they migrated towards the site. At approximately 20 minutes neutrophils increased in nearby blood vessels and within 30 minutes they had begun to transmigrate and move towards the LD site. By 45 minutes the transmigrated neutrophils had accumulated around the damage site. In contrast, few resident or vascular G184S neutrophil arrived, and the majority of those that did arrive, failed to migrate towards the site of injury or to accumulate (**Figure 5E** and **Supplementary Video 6**). Tracking individual neutrophils over the first hour shows the failure of G184S neutrophils to migrate towards the site of injury or to accumulate (**Figures 5F, G** **)**. Both the transmigrated and residential G184S neutrophils exhibited less directional migration and moved more erratically than did the WT neutrophils. The WT cells moved towards the site of LD while G184S cells often ignored the site and moved rapidly away (**Supplementary Figures 6E, F** **)**. These results show that *in vivo* both tissue resident and transmigrated G184S neutrophils have a poor sense of direction consistent with a chemotaxis defect.

### G184S Neutrophils Fragment and Aggregate in Liver Sinusoids Following Concanavalin A Administration

**Figure 6:**
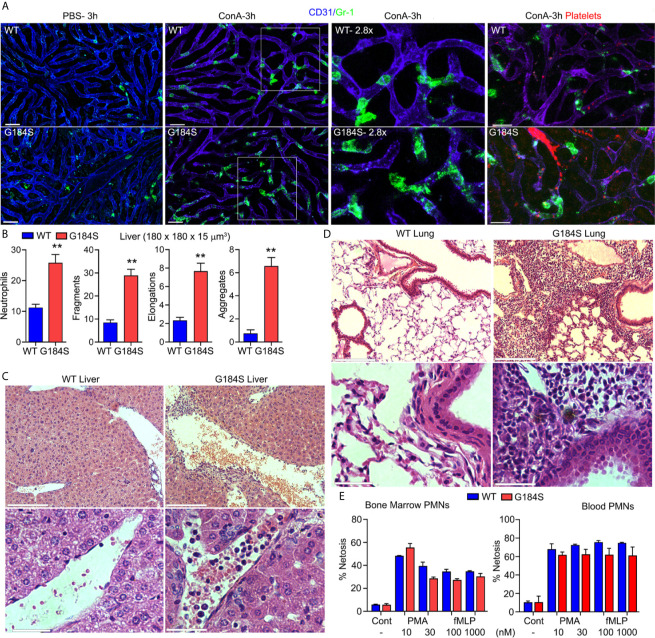
G184S neutrophils fragment and aggregate in liver sinusoids following concanavalin A (ConA) administration. **(A)** Representative confocal IVM images of *in vivo* WT (top panels) *vs* G184S (bottom panels) neutrophil behaviors/characteristics in the liver after 3 hours of ConA (i.v.) treatment. Images of PBS- (i.v.) treated control mice (right) compared to ConA-treated mice (middle) shows Gr-1 immunostained neutrophils (green) migrating within the CD31 immunostained liver sinusoids (blue). Areas outlined by white squares are shown magnified in the adjacent right panels. Scale bar = 30 µm. Far right, representative confocal IVM images of *in vivo* WT (top) *vs* G184S (bottom) platelet – neutrophil interactions and platelet behaviors/characteristics in the liver after 3 hours of ConA treatment. Both Gr-1 stained neutrophils (green) and GP1bβ labeled platelets (red) are observed within the CD31 immunostained liver sinusoids (violet). Scale bar = 20 µm. **(B)** Quantifications of neutrophil numbers, fragments, elongations, and aggregations within a 180 x 180 x 15 µm^3^ sections from WT or G184S BM reconstituted mice treated with ConA. **(C)** Representative liver images from histopathological examination comparing a WT to a G184S BM reconstituted mouse 3 hours after ConA injection. 10X (top panels) *vs* 40X (bottom panels) magnification. 10X, scale bar = 150 µm; 40X, scale bar = 30 µm. **(D)** Representative lung images from histopathological examination comparing WT to G184S BM reconstituted mouse 3 hours after ConA. 10X (top panels) *vs* 40X (bottom panels) magnification. 10X, scale bar = 150 µm; 40X, scale bar = 30 µm. **(E)** Quantifications of *ex vivo* NET-formation assays from isolated WT *vs* G184S BM (left graph) and blood (right graph) neutrophils stimulated with PMA or increasing doses of fMLP. All results are from WT (n = 3) and G184S (n = 3) BM reconstituted mice for each study. Statistics: data are means ± SEMs, then analyzed using unpaired Student’s *t*-test comparing G184S with WT. **p < 0.005.

In mice the inability of aging neutrophils to enter tissues for clearance predispose the mice to thrombo-inflammation and death (3). The poor transmigration and the aberrant aging of the G184S neutrophils prompted us to investigate whether the G184S neutrophils might cause or exacerbate vascular injury. The initial pathologic assessment of the lungs and liver in the WT and G184S BM reconstituted mice was unrevealing except for mild histologic evidence of hepatitis in both groups and an excess of neutrophils in the G184S mice organs (data not shown). To assess the liver vasculature *in vivo* we used intravital microscopy. We injected fluorescently labeled Gr-1 and CD31 antibodies as previously. PBS injected G184S BM reconstituted mice had more neutrophils in their liver sinusoids and a few more neutrophil fragments, but otherwise did not differ from the controls (**Figure 6A** and **Supplementary Figures 7A, B**). Next, we treated the mice with Concanavalin A (ConA), which triggers inflammatory changes in the liver vascular bed (37). ConA can also trigger neutrophil extracellular trap formation, a potential contributing factor to vascular damage (38). We administered a sublethal dose (2.5 mg/kg) intravenously and imaged 3-4 hours later. The G184S BM reconstituted mice suffered severe pulmonary distress during the later stages of the imaging (5 of 5 mice), while the WT BM reconstituted mice tolerated the procedure. Intravital microscopy revealed increased numbers of neutrophils in the liver sinusoids of the WT and G184S BM reconstituted mice, but more so in G184S BM reconstituted mice **(** **Figure 6A** and **Supplementary Video 7**). Furthermore, more neutrophil fragments and aggregates were present in the G184S BM reconstituted mice liver sinusoids (**Figure 6B**). Since neutrophils scan for activated platelets to initiate inflammation, we also imaged platelets in liver sinusoids of the ConA treated mice, which revealed platelet clots and aggregates, more prevalent in the G184S BM reconstituted mice (**Figure 6A**, **Supplementary Figure 7C** and **Supplementary Video 8**).

Next, we repeated the pathologic analysis following the ConA treatment. This revealed that the G184S livers had more liver sinusoid neutrophils and evidence of perivasculitis while similarly treated WT mice lacked the perivasculitis (**Figure 6C**). Slides prepared from the ConA treated G184S BM reconstituted mouse lungs revealed numerous neutrophils in the lung vasculature along with evidence of ongoing vascular inflammation, not observed in the controls (**Figure 6D**). This may explain the increased pulmonary distress noted in the ConA treated G184S BM reconstituted mice. The increased neutrophil fragmentation *in vivo* and apparent neutrophil extracellular traps in liver the sinusoids suggested that the G184S neutrophils may have an increased propensity to form extracellular traps (**Supplementary Video 9** **)**. To test this possibility BM and blood neutrophils isolated from BM chimera mice were investigated to assess extracellular traps formation before and after treatment with phorbol myristate acetate (PMA) or with fMLP. Both PMA and fMLP trigger extracellular trap formation by stimulating NADPH oxidase to form reactive oxygen species. However, these assays did not reveal any intrinsic difference between the WT and G184S blood and BM neutrophils (**Figure 6E**).

## Discussion

The G184S point mutation in the Gα_i2_ coding region disrupts the interaction between Gα_i2_ and RGS proteins. The failure of RGS proteins to bind Gα_i2_ slows the rate at which Gα_i2_-GTP returns to a GDP bound state. This has several consequences. First, the normal suppression of basal signaling provided by RGS proteins does not occur. Second, following GPCR triggered nucleotide exchange Gα_i2_-GTP can engage downstream effectors for longer durations. Furthermore, Gα_i2_-GTP does not recombine with freed G_β*γ*_ subunits, allowing them to persistently activate their own downstream effectors. This prolonged effector engagement by Gα_i_-GTP and free G_β*γ*_ subunits typically leads to hyperactivation of downstream signaling pathways (11, 39). Third, following ligand exposure the effective concentration of heterotrimeric Gα_i2_ available for GPCR activation declines. This can adversely affect the kinetics of the signal transduction pathways (21). Cells including neutrophils that lack an individual RGS protein typically exhibit enhanced GPCR signaling (11, 14, 40, 41). Furthermore, studies of the G184S mice have largely reported augmented GPCR-linked Gα_i_ signaling (42), yet past studies examining leukocyte chemoattractant receptors have largely reported diminished signaling (17, 21, 43). In the present study, we found that neutrophils, which carry the Gα_i2_ G184S mutation exhibit a complex phenotype with features of both hyperactive and hypoactive Gα_i_ signaling, which overlap with the phenotypes noted with Gα_i_ gain- or a loss- of function mutations.

A gain-of function mutation in a heterotrimeric Gα subunit typically results from the loss or diminution of its intrinsic GTPase activity. The Gα subunits remain persistently GTP bound unable to combine with Gβ*γ* subunits, allowing both to engage downstream effectors. For example, a rat Gα_i2_ protein with a T182A mutation releases GDP more rapidly and hydrolyzes GTP more slowly than does the wild type protein, thereby prolonging its GTP bound status (44). While the nearby G184S mutation does not affect GDP release, nor change the intrinsic Gα_i_ GTP hydrolysis rate; like the T182A mutation, it increases the duration that Gα_i2_ remains GTP bound. Consistent with an inappropriate or persistent engagement of downstream effectors, the G184S neutrophils have elevated basal intracellular calcium levels, increased basal cell motility, increased spontaneous spreading on ICAM-1 coated plates, decreased basal pEzrin levels, and an altered cell morphology. These base line changes likely reflect the failure of RGS proteins to suppress ligand-independent, and low-level, spurious ligand-dependent GPCR signaling. A major functional role for neutrophil RGS proteins may be to suppress background noise in chemoattractant receptor signaling pathways, thereby keeping the cell poised to respond to a chemoattractant signal. A similar role for RGS protein/Gα_i2_ interactions has been suggested in platelets, where they help provide a threshold for platelet activation (45). Compared to a Gα_i2_ G184S mutation, a T182A mutation would more severely disrupt GPCR-mediated Gα_i2_ signaling and likely result in a more adverse neutrophil phenotype. In fact, humans carrying a Gα_i2_ T182 mutation on one allele have innate immune defects and suffer recurrent sinopulmonary infections likely explained by neutrophil defects (46). No humans with a G184S Gα_i_ mutation have been reported. A G184S mutation on one allele may have a relatively mild phenotype, but homozygotes would likely have a severe multisystem phenotype. Similarly, a mouse or human carrying two Gα_i2_ T182A alleles would likely not be viable.

A loss-of-function of mutation in Gα_i2_ such as G203T sequesters Gβ*γ* subunits and GPCRs in their inactive conformations (47, 48). While no such mutation in Gα_i2_ has been reported in humans or engineered in mice, neutrophils with such a mutation would be expected to respond poorly to chemoattractants, resembling neutrophils that lack specific receptors or critical elements in the signaling pathway such as Gα_i_ (11). Neutrophils lacking Gα_i2_ exhibit impaired interstitial chemotaxis and poor accumulation at laser damage sites (35). A G184S mutation can mimic a loss-of-function mutation by limiting heterotrimer availability, by reducing GPCR/G-protein coupling, and by decreasing receptor availability. In neutrophils the G184S mutation caused a progressive loss of CXCR2 expression and CXCL1/2 triggered chemotaxis. Thus, the excessive engagement of effectors in the Gα_i2_ signaling pathway as neutrophils develop and age causes a progressive decline in CXCR2 expression and signaling. This was already evident even in BM neutrophils, which fail to mobilize properly to CXCL2. In contrast, CXCR4 expression in G184S neutrophils resembled that of WT cells and CXCR4 signaling in BM neutrophils remained largely intact even exaggerated at low ligand concentrations. However, eventually CXCL12 mediated chemotaxis declined and splenic neutrophil migrated poorly despite their adequate receptor expression. Thus, CXCR4 expression level is not sensitive to hyperactive Gα_i2_ signaling, but eventually the signal transduction pathway is affected. The reason for this progressive loss is unclear. Perhaps the inability to suppress low grade signals over time downregulates critical effectors in the chemotaxis signaling pathway. Further evidence of poor CXCR2 signaling in peripheral G184S neutrophils was their poor adherence to vascular endothelium *in vivo* and their inability to cross endothelial borders. This is despite their increased propensity to spread on ICAM-1 coated plates *in vitro* and it underscores the necessity of finely regulated Gα_i2_ mediated integrin activation for neutrophil adhesion to endothelial cells under flow for eventual neutrophil transmigration. Additional evidence of poor chemoattract receptor function, the G184S neutrophil that managed to transmigrate exhibit difficulties in maintaining a leading edge and following endogenous chemoattractant gradients.

The severe defects in neutrophil trafficking arise from the impact of the G184S Gα_i2_ mutation on chemoattractant receptor signaling pathways, however, less clear are the causes of the neutrophil fragmentation and vascular inflammation noted after treating the G184S BM reconstituted mice with ConA. We found no intrinsic difference in BM and blood neutrophil extracellular trap formation in response to PMA or to fMLP arguing that an increased propensity to form extracellular traps likely does not explain the ongoing vascular inflammation. Another possibility is abnormal neutrophil aging in these mice. Normal neutrophil aging begins after neutrophils leave the BM. Eventually aged neutrophils migrate into healthy tissues, a process termed clearance. This helps protect the vascular system from neutrophil mediated thrombo-inflammation (3, 49). Due to their severe TEM defect, the G184S mice neutrophils accumulate in both the lungs and liver vasculature. The TEM defect interferes both with the recruitment of neutrophils to inflammatory sites, but also with normal neutrophil clearance, thereby explaining the neutrophil overload. The persistent presence of aged neutrophils in the lung and liver blood vessels in the setting of an inflammatory insult may damage blood vessels and contributed to the ongoing thrombo-inflammation noted in the G184S mice following ConA treatment. However, we cannot exclude a role for abnormal platelet function as a contributing factor since the platelets in the G184S BM reconstituted mice also carry the G184S Gα_i2_ alleles (45). Also complicating the analysis of neutrophil aging in the G184S mice is the overlap between an “activated” and an aged phenotype. Further transcriptional studies of the neutrophil aging program in these mice is planned.

Our studies underscore two essential functions for RGS proteins in neutrophils. First, they suppress low grade environmental signals maintaining a low basal level of activity in Gα_i_-linked signaling pathways. This keeps the cells ready and able to respond to chemoattractant signals. Second, they help coordinate both the magnitude and duration of signals generated through Gα_i_-linked signaling. Interrupting these essential functions in neutrophils disrupts neutrophil homeostasis, trafficking, and function. Pharmaceutically targeting all Gα_i_/RGS interactions is likely to severely disable neutrophils. A more finely tuned intervention will require a better understanding of how individual RGS proteins cooperate to regulate chemoattractant receptor signaling.

## Data Availability Statement

Requests to access the datasets should be directed to jkehrl@niaid.nih.gov.

## Ethics Statement

The animal study was reviewed and approved by NIAID Animal and Use Committee.

## Author Contributions

SY, OK, JSK, and CP performed the imaging experiments. I-YH performed the flow cytometry and migration experiments, and managed the mice. SY, I-YH, and JKe designed the experiments. SY and JKe wrote the manuscript and JKe oversaw the project. All authors contributed to the article and approved the submitted version.

## Funding

This work was supported by Intramural Research Program of National Institute of Allergy and Infectious Diseases.

## Disclaimer

The content is solely the responsibility of the authors and does not necessarily represent the official views of the National Institutes of Health.

## Conflict of Interest

The authors declare that the research was conducted in the absence of any commercial or financial relationships that could be construed as a potential conflict of interest.

## References

[1] Eash KJ Greenbaum AM Gopalan PK Link DC. CXCR2 and CXCR4 Antagonistically Regulate Neutrophil Trafficking From Murine Bone Marrow. J Clin Invest (2010) 120(7):2423–31. 10.1172/JCI41649 PMC2898597 20516641

[2] Liu Q Li Z Gao JL Wan W Ganesan S McDermott DH. CXCR4 Antagonist AMD3100 Redistributes Leukocytes From Primary Immune Organs to Secondary Immune Organs, Lung, and Blood in Mice. Eur J Immunol (2015) 45(6):1855–67. 10.1002/eji.201445245 PMC4461468 25801950

[3] Adrover JM Del Fresno C Crainiciuc G Cuartero MI Casanova-Acebes M Weiss LA. A Neutrophil Timer Coordinates Immune Defense and Vascular Protection. Immunity (2019) 51(5):966–7. 10.1016/j.immuni.2019.11.001 31747583

[4] Adrover JM Aroca-Crevillen A Crainiciuc G Ostos F Rojas-Vega Y Rubio-Ponce A. Programmed ‘Disarming’ of the Neutrophil Proteome Reduces the Magnitude of Inflammation. Nat Immunol (2020) 21(2):135–44. 10.1038/s41590-019-0571-2 PMC7223223 31932813

[5] Adrover JM Nicolas-Avila JA Hidalgo A. Aging: A Temporal Dimension for Neutrophils. Trends Immunol (2016) 37(5):334–45. 10.1016/j.it.2016.03.005 27083489

[6] Hidalgo A Chilvers ER Summers C Koenderman L. The Neutrophil Life Cycle. Trends Immunol (2019) 40(7):584–97. 10.1016/j.it.2019.04.013 31153737

[7] Furze RC Rankin SM. Neutrophil Mobilization and Clearance in the Bone Marrow. Immunology (2008) 125(3):281–8. 10.1111/j.1365-2567.2008.02950.x PMC2669132 19128361

[8] Kehrl JH. Chemoattractant Receptor Signaling and the Control of Lymphocyte Migration. Immunol Res (2006) 34(3):211–27. 10.1385/IR:34:3:211 16891672

[9] Cho H Kehrl JH. Regulation of Immune Function by G Protein-Coupled Receptors, Trimeric G Proteins, and RGS Proteins. Prog Mol Biol Transl Sci (2009) 86:249–98. 10.1016/S1877-1173(09)86009-2 20374719

[10] Kamp ME Liu Y Kortholt A. Function and Regulation of Heterotrimeric G Proteins During Chemotaxis. Int J Mol Sci (2016) 17(1):90–104. 10.3390/ijms17010090 PMC4730333 26784171

[11] Kehrl JH. The Impact of RGS and Other G-protein Regulatory Proteins on Galphai-mediated Signaling in Immunity. Biochem Pharmacol (2016) 114:40–52. 10.1016/j.bcp.2016.04.005 27071343 PMC4993105

[12] George T Bell M Chakraborty M Siderovski DP Giembycz MA Newton R. Protective Roles for RGS2 in a Mouse Model of House Dust Mite-Induced Airway Inflammation. PloS One (2017) 12(1):e0170269. 10.1371/journal.pone.0170269 28107494 PMC5249169

[13] George T Chakraborty M Giembycz MA Newton R. A Bronchoprotective Role for Rgs2 in a Murine Model of Lipopolysaccharide-Induced Airways Inflammation. Allergy Asthma Clin Immunol (2018) 14:40. 10.1186/s13223-018-0266-5 30305828 PMC6166284

[14] Chan EC Ren C Xie Z Jude J Barker T Koziol-White CA. Regulator of G Protein Signaling 5 Restricts Neutrophil Chemotaxis and Trafficking. J Biol Chem (2018) 293(33):12690–702. 10.1074/jbc.RA118.002404 PMC6102127 29929985

[15] Lan KL Sarvazyan NA Taussig R Mackenzie RG DiBello PR Dohlman HG. A Point Mutation in Galphao and Galphai1 Blocks Interaction With Regulator of G Protein Signaling Proteins. J Biol Chem (1998) 273(21):12794–7. 10.1074/jbc.273.21.12794 9582306

[16] Huang X Fu Y Charbeneau RA Saunders TL Taylor DK Hankenson KD. Pleiotropic Phenotype of a Genomic Knock-in of an RGS-insensitive G184s Gnai2 Allele. Mol Cell Biol (2006) 26(18):6870–9. 10.1128/MCB.00314-06 PMC1592866 16943428

[17] Cho H Kamenyeva O Yung S Gao JL Hwang IY Park C. The Loss of RGS protein-Galpha(i2) Interactions Results in Markedly Impaired Mouse Neutrophil Trafficking to Inflammatory Sites. Mol Cell Biol (2012) 32(22):4561–71. 10.1128/MCB.00651-12 PMC3486189 22966200

[18] Yu YR O’Koren EG Hotten DF Kan MJ Kopin D Nelson ER. A Protocol for the Comprehensive Flow Cytometric Analysis of Immune Cells in Normal and Inflamed Murine Non-Lymphoid Tissues. PloS One (2016) 11(3):e0150606. 10.1371/journal.pone.0150606 26938654 PMC4777539

[19] Uhl B Vadlau Y Zuchtriegel G Nekolla K Sharaf K Gaertner F. Aged Neutrophils Contribute to the First Line of Defense in the Acute Inflammatory Response. Blood (2016) 128(19):2327–37. 10.1182/blood-2016-05-718999 PMC5122310 27609642

[20] Martin C Burdon PC Bridger G Gutierrez-Ramos JC Williams TJ Rankin SM. Chemokines Acting Via CXCR2 and CXCR4 Control the Release of Neutrophils From the Bone Marrow and Their Return Following Senescence. Immunity (2003) 19(4):583–93. 10.1016/S1074-7613(03)00263-2 14563322

[21] Hwang IY Park C Harrison K Kehrl JH. Normal Thymocyte Egress, T Cell Trafficking, and CD4(+) T Cell Homeostasis Require Interactions Between RGS Proteins and Galphai2. J Immunol (2017) 198(7):2721–34. 10.4049/jimmunol.1601433 PMC5360501 28235863

[22] Park C Kehrl JH. An Integrin/MFG-E8 Shuttle Loads HIV-1 Viral-Like Particles Onto Follicular Dendritic Cells in Mouse Lymph Node. Elife (2019) 8:1–26. 10.7554/eLife.47776 PMC6901335 31793433

[23] Lo Celso C Lin CP Scadden DT. In Vivo Imaging of Transplanted Hematopoietic Stem and Progenitor Cells in Mouse Calvarium Bone Marrow. Nat Protoc (2011) 6(1):1–14. 10.1038/nprot.2010.168 21212779 PMC3382040

[24] Yan SLS Hwang IY Kamenyeva O Kehrl JH. In Vivo F-Actin Filament Organization During Lymphocyte Transendothelial and Interstitial Migration Revealed by Intravital Microscopy. iScience (2019) 16:283–97. 10.1016/j.isci.2019.05.040 PMC6581778 31203185

[25] Grosse J Braun A Varga-Szabo D Beyersdorf N Schneider B Zeitlmann L. An EF Hand Mutation in Stim1 Causes Premature Platelet Activation and Bleeding in Mice. J Clin Invest (2007) 117(11):3540–50. 10.1172/JCI32312 PMC2040319 17965774

[26] Casanova-Acebes M Pitaval C Weiss LA Nombela-Arrieta C Chevre R AG N. Rhythmic Modulation of the Hematopoietic Niche Through Neutrophil Clearance. Cell (2013) 153(5):1025–35. 10.1016/j.cell.2013.04.040 PMC4128329 23706740

[27] Kamenyeva O Boularan C Kabat J Cheung GY Cicala C Yeh AJ. Neutrophil Recruitment to Lymph Nodes Limits Local Humoral Response to Staphylococcus Aureus. PloS Pathog (2015) 11(4):e1004827. 10.1371/journal.ppat.1004827 25884622 PMC4401519

[28] Tomay F Wells K Duong L Tsu JW Dye DE Radley-Crabb HG. Aged Neutrophils Accumulate in Lymphoid Tissues From Healthy Elderly Mice and Infiltrate T- and B-cell Zones. Immunol Cell Biol (2018) 96(8):831–40. 10.1111/imcb.12046 29603362

[29] Bronte V Pittet MJ. The Spleen in Local and Systemic Regulation of Immunity. Immunity (2013) 39(5):806–18. 10.1016/j.immuni.2013.10.010 PMC3912742 24238338

[30] Girbl T Lenn T Perez L Rolas L Barkaway A Thiriot A. Distinct Compartmentalization of the Chemokines CXCL1 and CXCL2 and the Atypical Receptor ACKR1 Determine Discrete Stages of Neutrophil Diapedesis. Immunity (2018) 49(6):1062–76 e6. 10.1016/j.immuni.2018.09.018 30446388 PMC6303217

[31] Redpath AN Francois M Wong SP Bonnet D Rankin SM. Two Distinct CXCR4 Antagonists Mobilize Progenitor Cells in Mice by Different Mechanisms. Blood Adv (2017) 1(22):1934–43. 10.1182/bloodadvances.2017006064 PMC5728142 29296840

[32] Pillay J Tregay N Juzenaite G Carlin LM Pirillo C Gaboriau DCA. Effect of the CXCR4 Antagonist Plerixafor on Endogenous Neutrophil Dynamics in the Bone Marrow, Lung and Spleen. J Leukoc Biol (2020) 107(6):1175–85. 10.1002/JLB.1MA0420-571RR 32374077

[33] Ley K Laudanna C Cybulsky MI Nourshargh S. Getting to the Site of Inflammation: The Leukocyte Adhesion Cascade Updated. Nat Rev Immunol (2007) 7(9):678–89. 10.1038/nri2156 17717539

[34] Woodfin A Voisin MB Beyrau M Colom B Caille D Diapouli FM. The Junctional Adhesion Molecule JAM-C Regulates Polarized Transendothelial Migration of Neutrophils In Vivo. Nat Immunol (2011) 12(8):761–9. 10.1038/ni.2062 PMC3145149 21706006

[35] Lammermann T Afonso PV Angermann BR Wang JM Kastenmuller W Parent CA. Neutrophil Swarms Require LTB4 and Integrins At Sites of Cell Death In Vivo. Nature (2013) 498(7454):371–5. 10.1038/nature12175 PMC3879961 23708969

[36] Ng LG Qin JS Roediger B Wang Y Jain R Cavanagh LL. Visualizing the Neutrophil Response to Sterile Tissue Injury in Mouse Dermis Reveals a Three-Phase Cascade of Events. J Invest Dermatol (2011) 131(10):2058–68. 10.1038/jid.2011.179 21697893

[37] Siegmund K Lee WY Tchang VS Stiess M Terracciano L Kubes P. Coronin 1 is Dispensable for Leukocyte Recruitment and Liver Injury in Concanavalin A-induced Hepatitis. Immunol Lett (2013) 153(1-2):62–70. 10.1016/j.imlet.2013.06.005 23856257

[38] Amulic B Knackstedt SL Abu Abed U Deigendesch N Harbort CJ Caffrey BE. Cell-Cycle Proteins Control Production of Neutrophil Extracellular Traps. Dev Cell (2017) 43(4):449–62.e5. 10.1016/j.devcel.2017.10.013 29103955

[39] Woodard GE Jardin I Berna-Erro A Salido GM Rosado JA. Regulators of G-protein-signaling Proteins: Negative Modulators of G-protein-coupled Receptor Signaling. Int Rev Cell Mol Biol (2015) 317:97–183. 10.1016/bs.ircmb.2015.02.001 26008785

[40] Balenga NA Jester W Jiang M Panettieri RA Jr. Druey KM. Loss of Regulator of G Protein Signaling 5 Promotes Airway Hyperresponsiveness in the Absence of Allergic Inflammation. J Allergy Clin Immunol (2014) 134(2):451–9. 10.1016/j.jaci.2014.01.019 PMC4119844 24666695

[41] Xie Z Chan EC Druey KM. R4 Regulator of G Protein Signaling (Rgs) Proteins in Inflammation and Immunity. AAPS J (2016) 18(2):294–304. 10.1208/s12248-015-9847-0 26597290 PMC4779105

[42] Neubig RR. Rgs-Insensitive G Proteins as In Vivo Probes of RGS Function. Prog Mol Biol Transl Sci (2015) 133:13–30. 10.1016/bs.pmbts.2015.04.010 26123300

[43] Hwang IY Park C Harrison K Boularan C Gales C Kehrl JH. An Essential Role for RGS Protein/Galphai2 Interactions in B Lymphocyte-Directed Cell Migration and Trafficking. J Immunol (2015) 194(5):2128–39. 10.4049/jimmunol.1401952 PMC4339488 25617475

[44] Nishina H Nimota K Kukimoto I Maehama T Takahashi K Hoshino S. Significance of Thr182 in the Nucleotide-Exchange and GTP-hydrolysis Reactions of the Alpha Subunit of GTP-binding Protein Gi2. J Biochem (1995) 118(5):1083–9. 10.1093/jb/118.5.1083 8749330

[45] Signarvic RS Cierniewska A Stalker TJ Fong KP Chatterjee MS Hess PR. Rgs/Gi2alpha Interactions Modulate Platelet Accumulation and Thrombus Formation At Sites of Vascular Injury. Blood (2010) 116(26):6092–100. 10.1182/blood-2010-05-283846 PMC3031394 20852125

[46] Lamborn IT Su HC Lenardo MJ Bhandoola A Cherry S Schwartzberg PL. The Genetic, Molecular, and Cellular Bases of Unidentified Primary Immunodeficiencies. Publicly Accessible Penn Dissertations (2016) 2016:160. 2410.

[47] Murray-Whelan R Reid JD Piuz I Hezareh M Schlegel W. The Guanine-Nucleotide-Binding Protein Subunit G Alpha I2 is Involved in Calcium Activation of Phospholipase A2. Effects of the Dominant Negative G Alpha I2 Mutant, [G203T]G Alpha i2, on Activation of Phospholipase A2 in Chinese Hamster Ovary Cells. Eur J Biochem (1995) 230(1):164–9. 10.1111/j.1432-1033.1995.tb20547.x 7601096

[48] Inoue S Hoshino S Kukimoto I Ui M Katada T. Purification and Characterization of the G203T Mutant Alpha I-2 Subunit of GTP-binding Protein Expressed in Baculovirus-Infected Sf9 Cells. J Biochem (1995) 118(3):650–7. 10.1093/oxfordjournals.jbchem.a124959 8690731

[49] Casanova-Acebes M Nicolas-Avila JA Li JL Garcia-Silva S Balachander A Rubio-Ponce A. Neutrophils Instruct Homeostatic and Pathological States in Naive Tissues. J Exp Med (2018) 215(11):2778–95. 10.1084/jem.20181468 PMC6219739 30282719

