# Supplementary material for: Unrestrained Gαi2 Signaling Disrupts Neutrophil Trafficking, Aging, and Clearance
Source: Front Immunol. 2021 May 31;12:679856. doi: 10.3389/fimmu.2021.679856 (PMC8202015; doi:10.3389/fimmu.2021.679856)
Supplement: Supplementary file 1 [file DataSheet_1.pdf]

Unrestrained $G\alpha_{i2}$ Signaling Disrupts Normal Neutrophil Trafficking, Aging, and Clearance

Serena Li-Sue Yan, Il-Young Hwang, Olena Kamenyeva, Juraj Kabat, Ji Sung Kim, Chung Park, and John H. Kehrl

Supplemental Information:

1. Supplementary Figures S1-S8 and Tables S1 & 2.
2. Supplementary Video legends 1-9
3. Reagent resource table
4. Flow cytometry instrumentation.

1. Supplement Figures and Tables

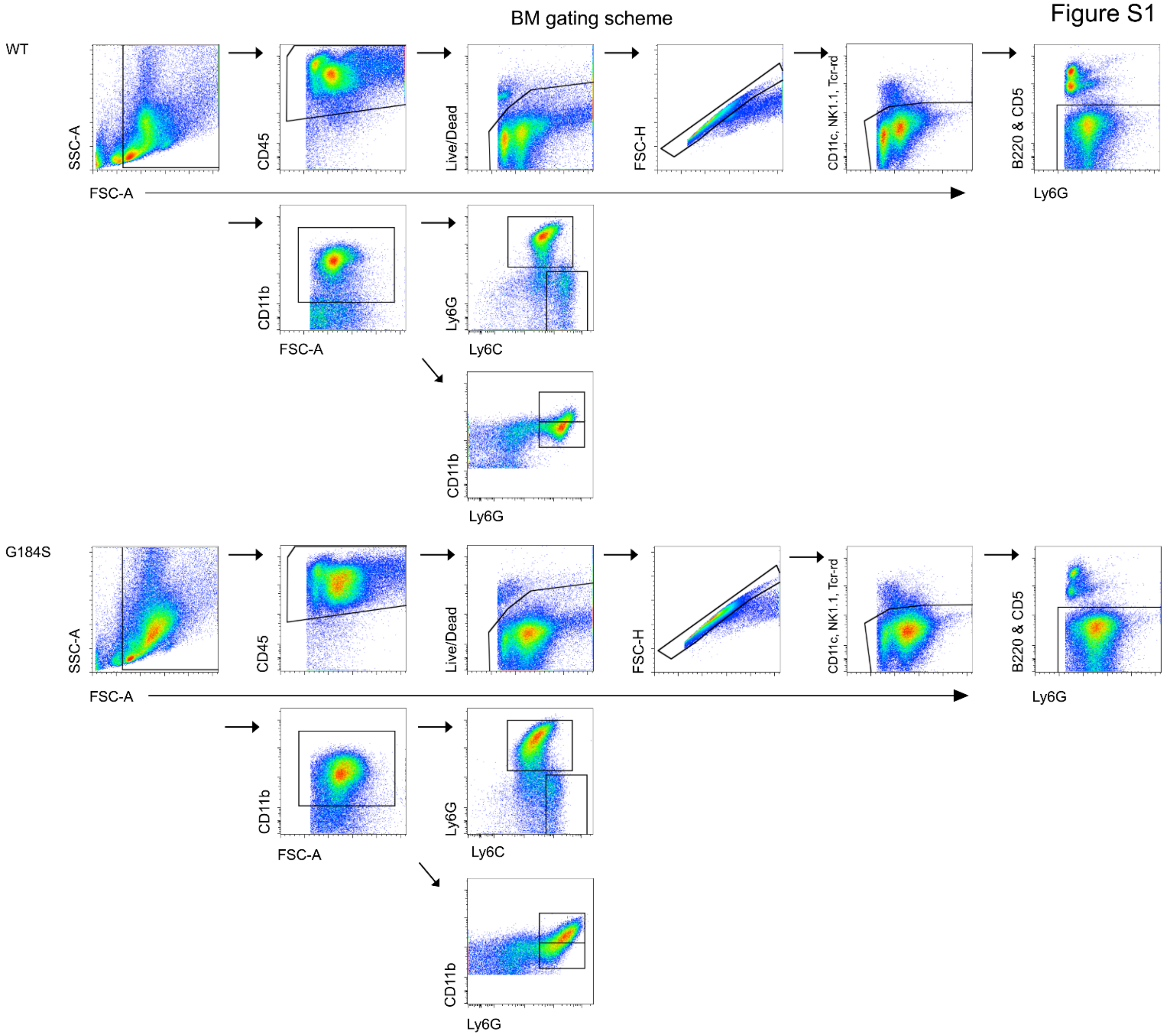

Supplementary Figure 1. Flow cytometry gating strategy for analysis of neutrophils from WT and G184S bone marrow.

Figure S2

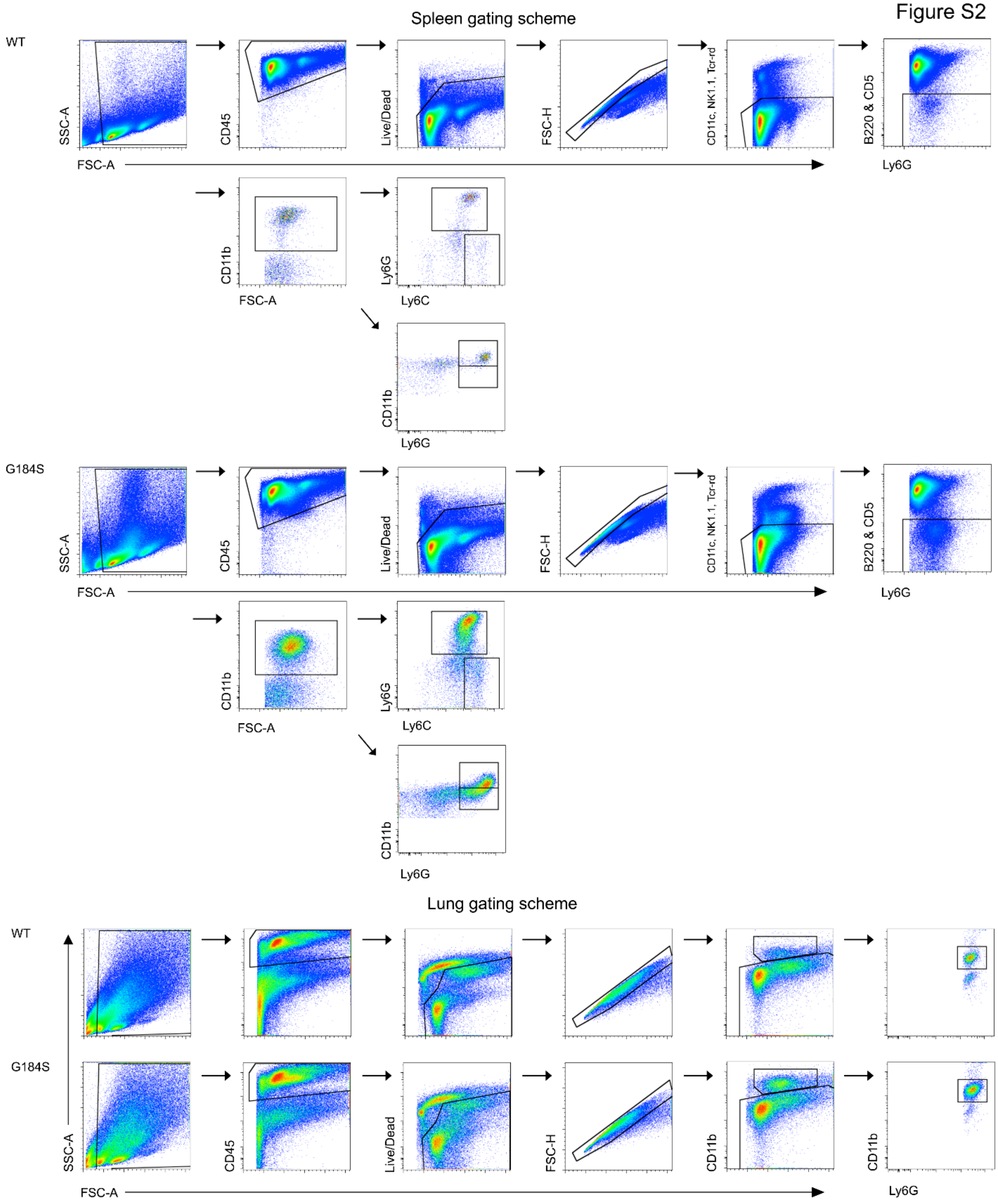

Supplementary Figure 2. Flow cytometry gating strategy for analysis of neutrophils from WT and G184S spleen (above) and lung (below).

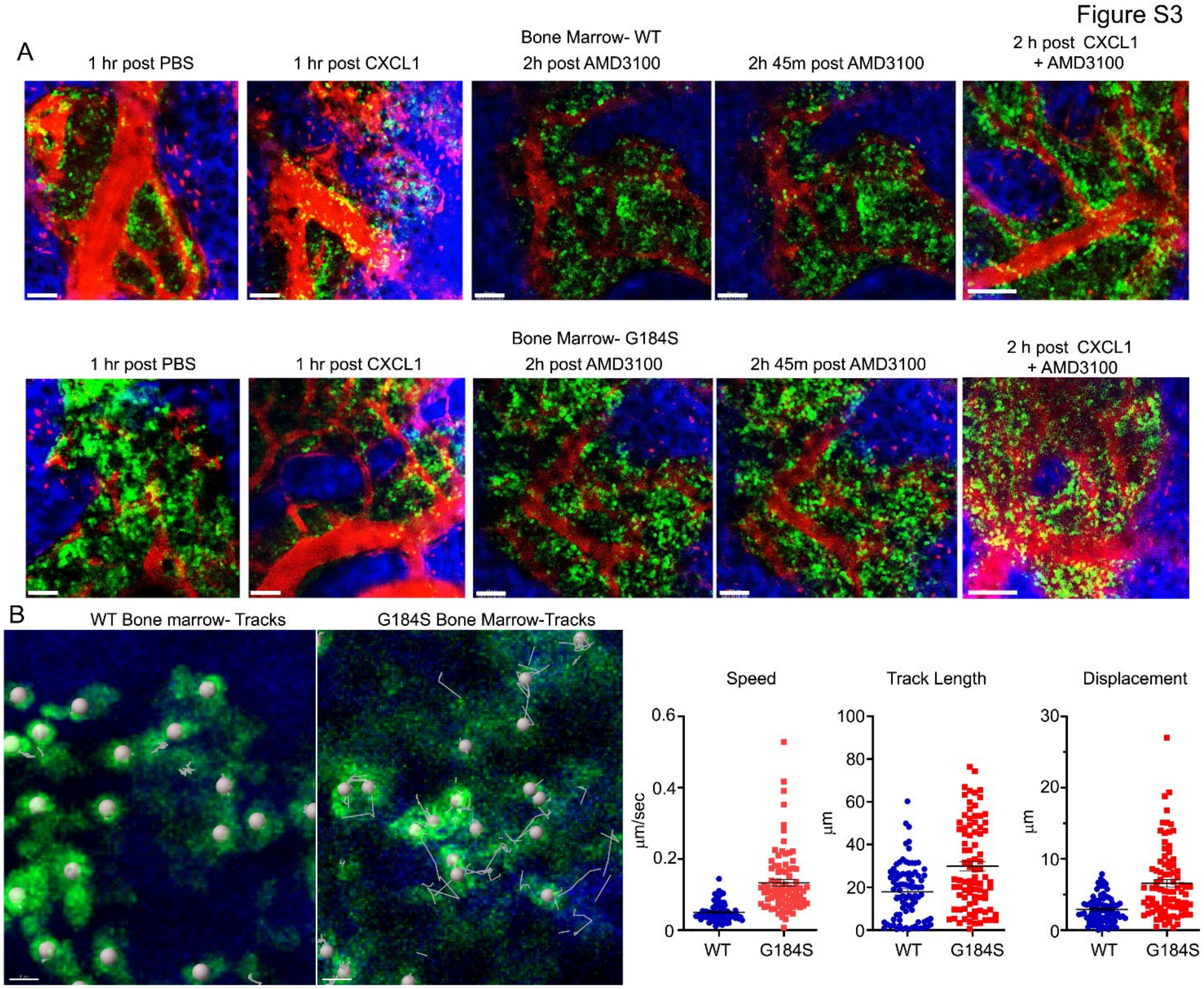

Supplementary Figure 3. Imaging neutrophils in the bone marrow in the presence or absence of intravenous CXCL1, AMD3100, or both. **(A)** 2P-IVM images of skull BM in WT vs G184S mice with LysM-GFP neutrophils (green), Evans Blue labeled blood vessels (red), and collagen (blue). Comparison of WT (top panels) to G184S (bottom panels) neutrophil BM localization and intravascular mobilization after either PBS (control), CXCL1, or AMD3100 injections (panels 1 – 4 from far left). Scale bar = 50 μm. Far right panels, 2 hrs after CXCL1 and AMD3100 injections. Scale bars = 100 μm. Images are representative of WT (n = 3) and G184S (n = 3) mice for each study. **(B)** 2 P-IVM images of skull BM in WT and G184S mice with LysM-GFP neutrophils (green) under basal conditions. The cells are overlaid with grey spheres designating their last location in a 10-minute imaging sequence (images captured every 10 seconds). Cell tracks over the previous 10 minutes are shown in grey. Scale bars = 8 μm. Many of the WT tracks are obscured as the cells remained stationary during the imaging period. Tracking parameters shown on the right were obtained from approximately 80 cells tracked over ten minutes. These results are representative of 3 experiments.

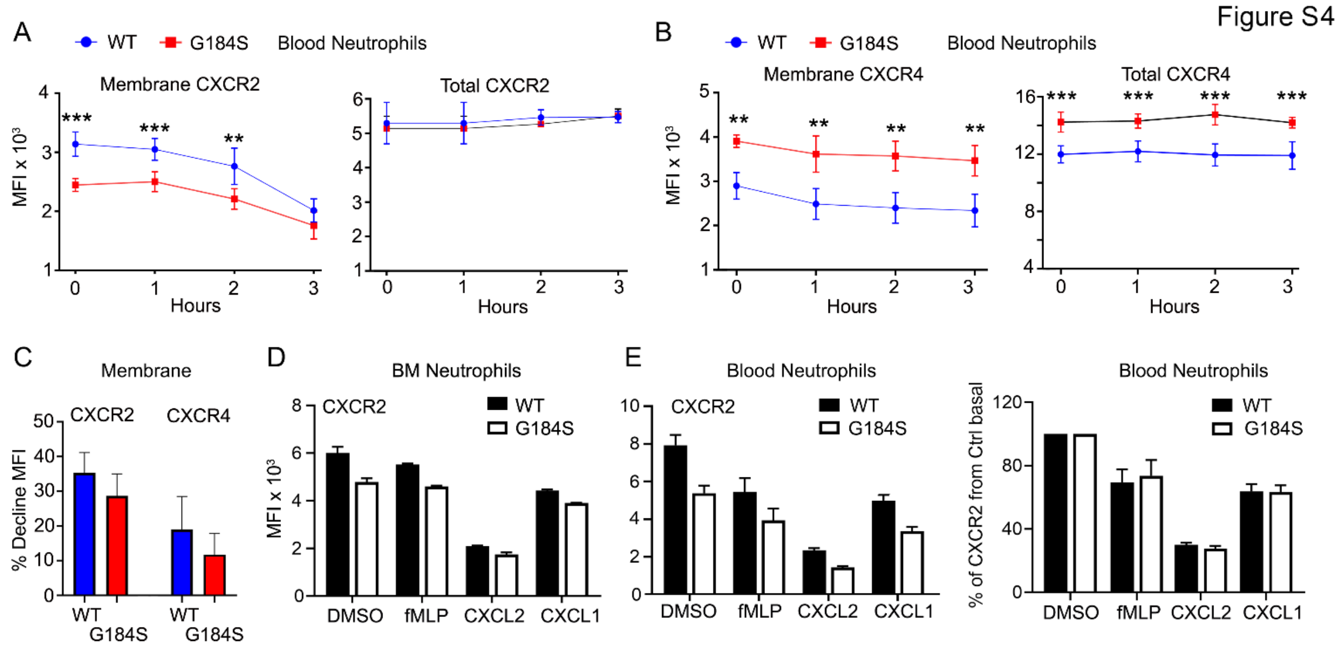

Supplementary Figure 4. CXCR4, CXCR2, and CD62L expression levels of WT vs G184S neutrophils. **(A)** Flow cytometry results measuring membrane (left) and total (right) CXCR2 expression levels of WT vs G184S blood neutrophils in KRH3955 treated mice over 3 hours. **(B)** Results of membrane (left) and total (right) CXCR4 expression levels of WT vs G184S blood neutrophils in KRH3955 treated mice over 3 hours measured by flow cytometry. **(C)** Flow cytometry data showing the final % decline from basal control of membrane CXCR2 and CXCR4 of WT vs G184S blood neutrophils by 3 hours post KRH3955 treatment. **(D, E)** Flow cytometry results measuring membrane CXCR2 expression levels of WT vs G184S neutrophils from BM, D, and blood, E, exposed to fMLP, CXCL2, and CXCL1 for 30 minutes. Final % decline from basal control of membrane CXCR2 of WT vs G184S blood neutrophils post fMLP, CXCL2, and CXCL1 treatment measured by flow cytometry. All results are from n = 3 separate experiments done in triplicates using 1:1 WT:G184S BM reconstituted mice for each study. Statistics: data are means ± SEMs, then analyzed using unpaired Student's *t*-test comparing G184S with WT. $^{**}p < 0.005$ and $^{***}p < 0.0005$.

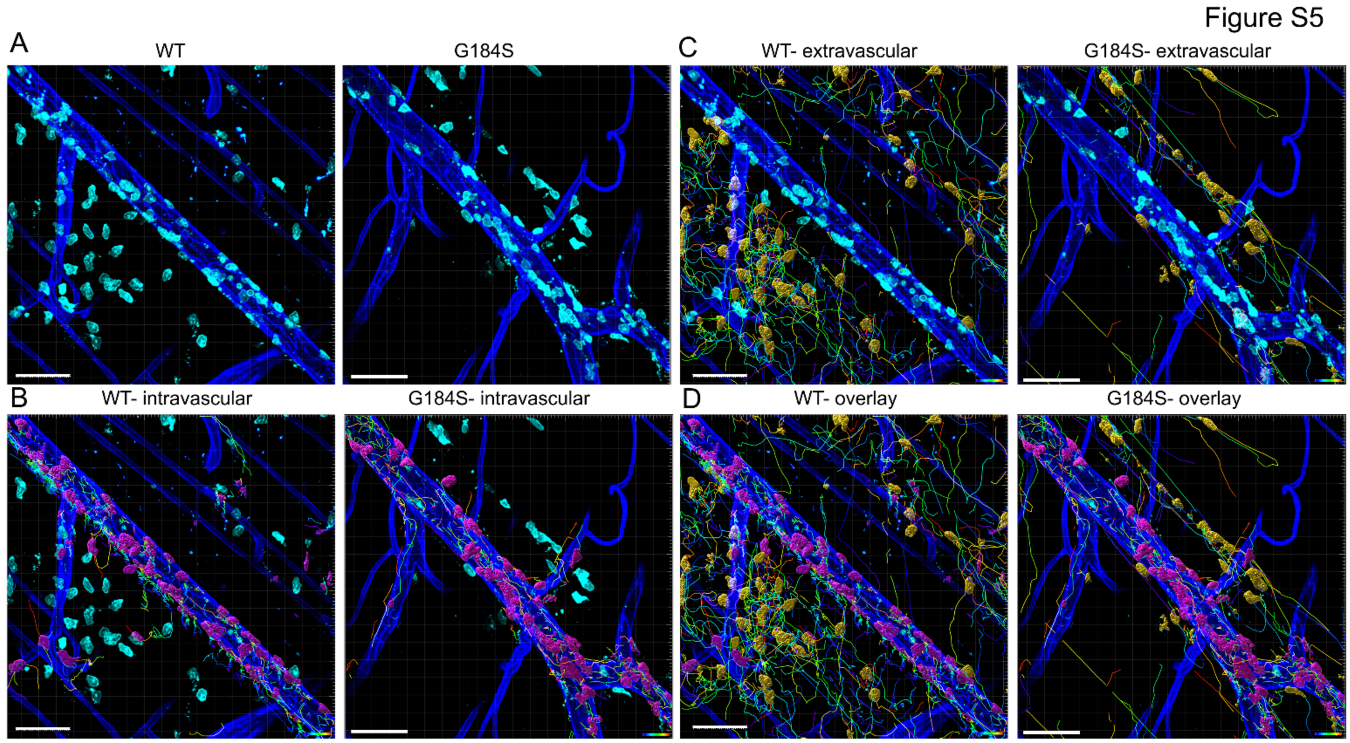

Supplementary Figure 5. Distinguishing between intravascular and transmigrated neutrophils. **(A)** Confocal IVM images showing *in vivo* WT (left) vs G184S (right) neutrophil migration in cremaster muscle after 90 minutes of IL-1β (i.v.) and AMD3100 (i.p.) treatment. Neutrophils (cyan) and blood vessels (blue) are visualized by immunostaining with fluorescently labeled Gr-1 and CD31 antibodies, respectively. **(B,C)** Intravascular (attached) or transmigrated extravascular (free) neutrophils identified via Imaris software analysis. Using Imaris surface tool, the intravascular neutrophils are set to associate with CD31 staining (attached) and created a new surface (magenta) as identifier, shown in B. Transmigrated neutrophils are set to have no association with CD31 staining (free) and created a new surface (yellow) as identifier, shown in C. **(D)** Overlay of extravascular and intravascular time dependent tracks. After the 2 surfaces are created, neutrophils can be tracked and divided into separate groups (attached vs free) for data analysis. Scale bars = 60 μm.

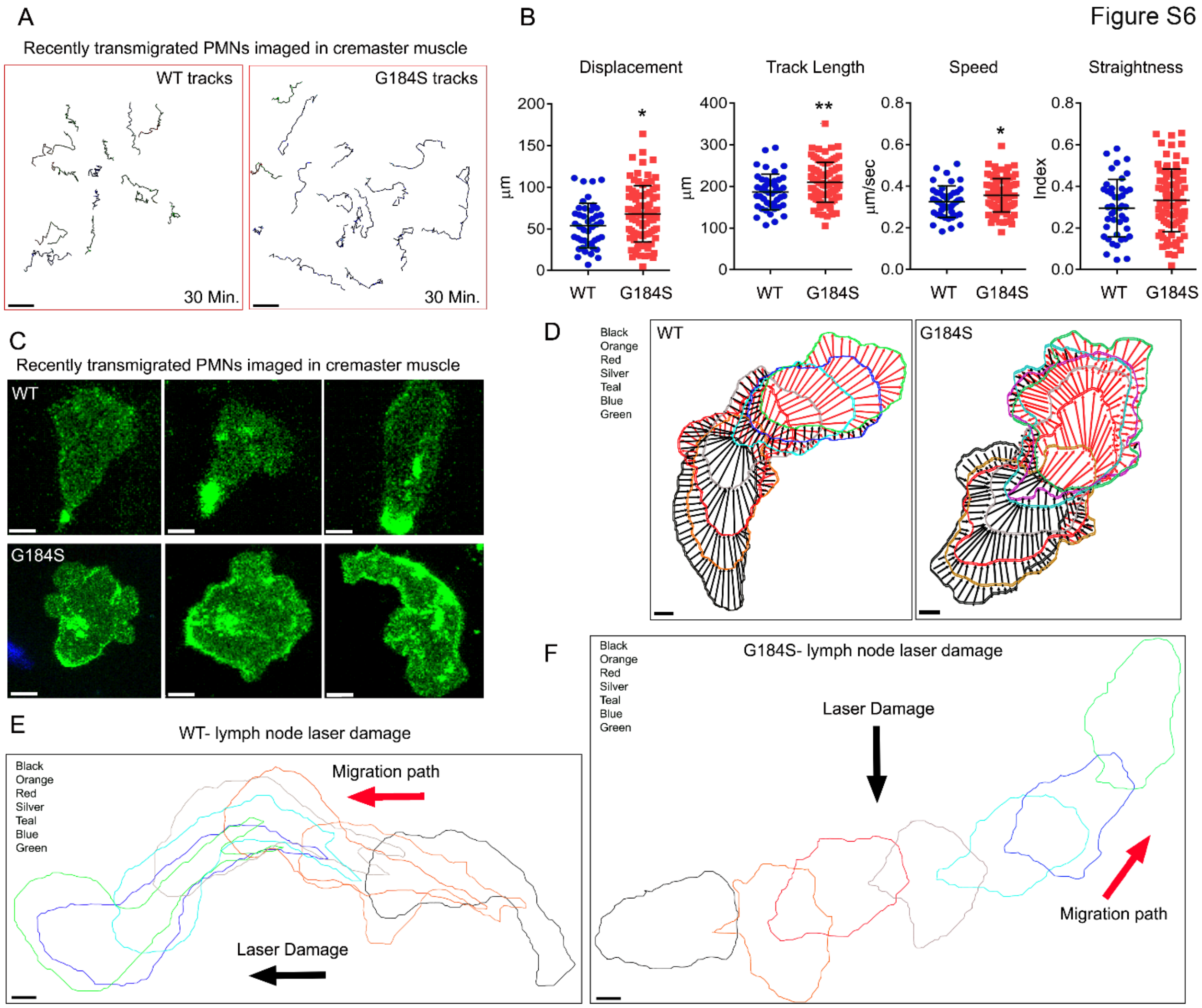

Supplementary Figure 6. *In vivo* interstitial migration of WT versus G184S neutrophils. **(A)** In Vivo Tracks WT (left) vs G184S (right) neutrophil interstitial migration in the cremaster muscle after IL-1β (i.v.) and AMD3100 (i.p.) treatment. Neutrophils tracked for ~30 minutes. Scale bar = 30 µm. **(B)** Displacement, track length, speed, and straightness of interstitial WT vs G184S neutrophils over 30 minutes. **(C)** Confocal IVM images show morphology of individual Gr-1 immunostained WT (top panels) vs G184S (bottom panels) neutrophil migrating in the cremaster muscle interstitium. Scale bars = 3 µm. **(D)** Images show *in vivo* leading and trailing edge organization of a single WT (left) vs single G184S (right) neutrophil during interstitial migration. Position changes of the neutrophils (1 frame per 25 second) are represented via different colors, with "Black" as the start. Scale bars = 3 µm. **(E, F)** Images of a WT (E) vs G184S (F) neutrophil interstitial migration in mice inguinal lymph node (LN) after laser damage. The black arrows indicate direction of site of laser damage, and the red arrows show the direction of neutrophil migration path. Neutrophil position changes (1 frame per 25 second) are represented via different colors, with "Black" as the start. Scale bars = 3 µm. (A, B, C, D) Results are from WT (n = 5) and G184S (n = 5) BM reconstituted mice with 90 min treatment of IL-1β and AMD3100. Results in panel E are representative from the analysis of WT (n = 3) and G184S (n = 3) BM reconstituted mice after 2P laser damage within the LN. Statistics: data are means ± SEMs, then analyzed using unpaired Student's *t*-test comparing G184S with WT. * $p < 0.05$ and ** $p < 0.005$.

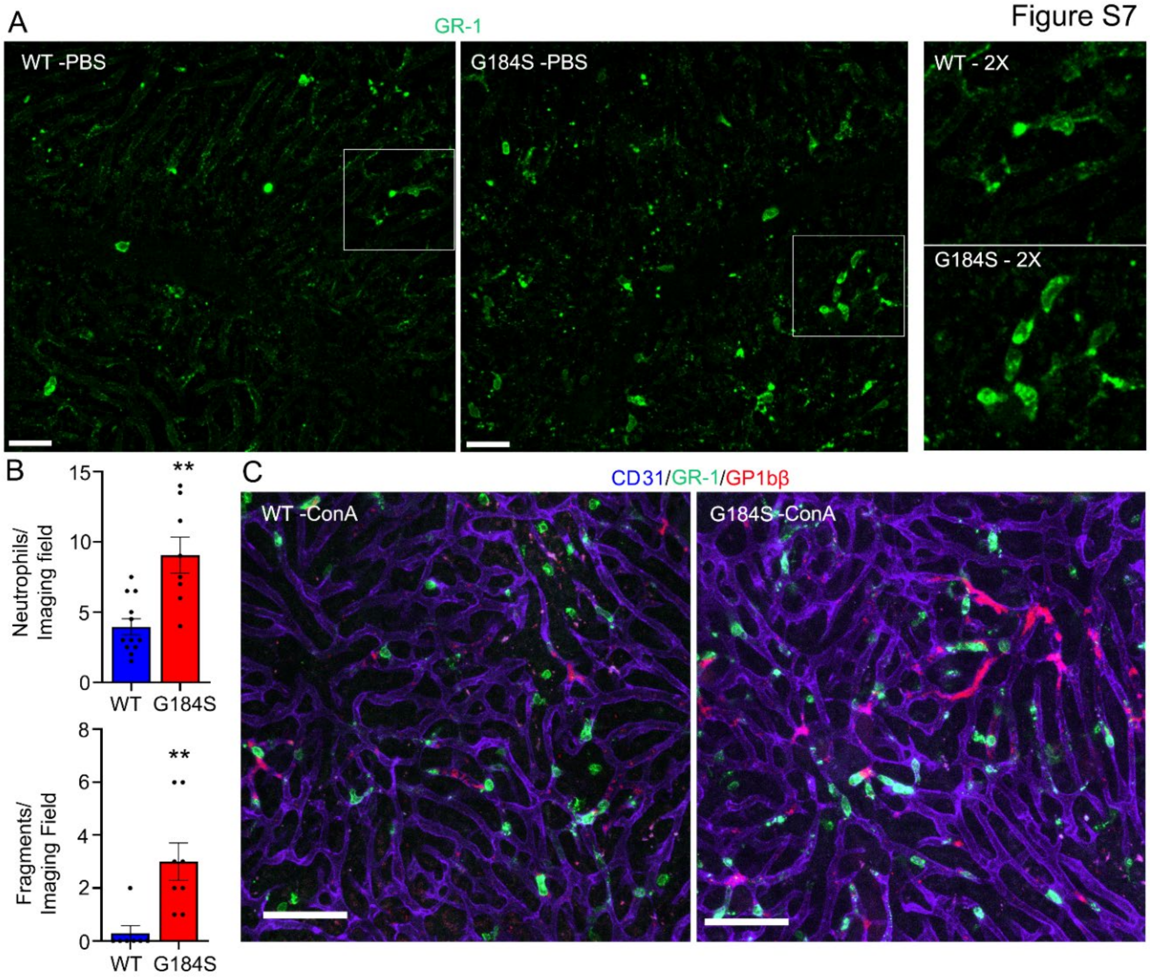

Supplementary Figure 7. G184S neutrophil and platelet imaging in liver sinusoids. (A) Full wide field confocal IVM of liver from WT (left) and G184S bone marrow reconstituted mice (right). Only green channel is shown to illustrate GR-1 postive neutrophils and neutrophil fragment. Scale bar = 30 µm. (B) Quantification of neutrophil numbers and fragments within a 180 x 180 x 15 µm sections from WT or G184S BM reconstituted mice treated with PBS. All results are from WT (n = 3) and G184S (n = 3) BM reconstituted mice for each study. Statistics: data are means + SEMs, then analyzed using unpaired Student's t-test comparing G184S with WT. $^{**}p < 0.005$. (C) Representative full wide-field confocal IVM images of *in vivo* WT (left panel) vs G184S (right panel) platelet – neutrophil interactions and platelet behaviors/characteristics in the liver after 3 hours of ConA treatment. Both Gr-1 stained neutrophils (green) and GP1bβ labeled platelets (red) are observed within the CD31 immunostained liver sinusoids (violet). Scale bar = 70 µm.

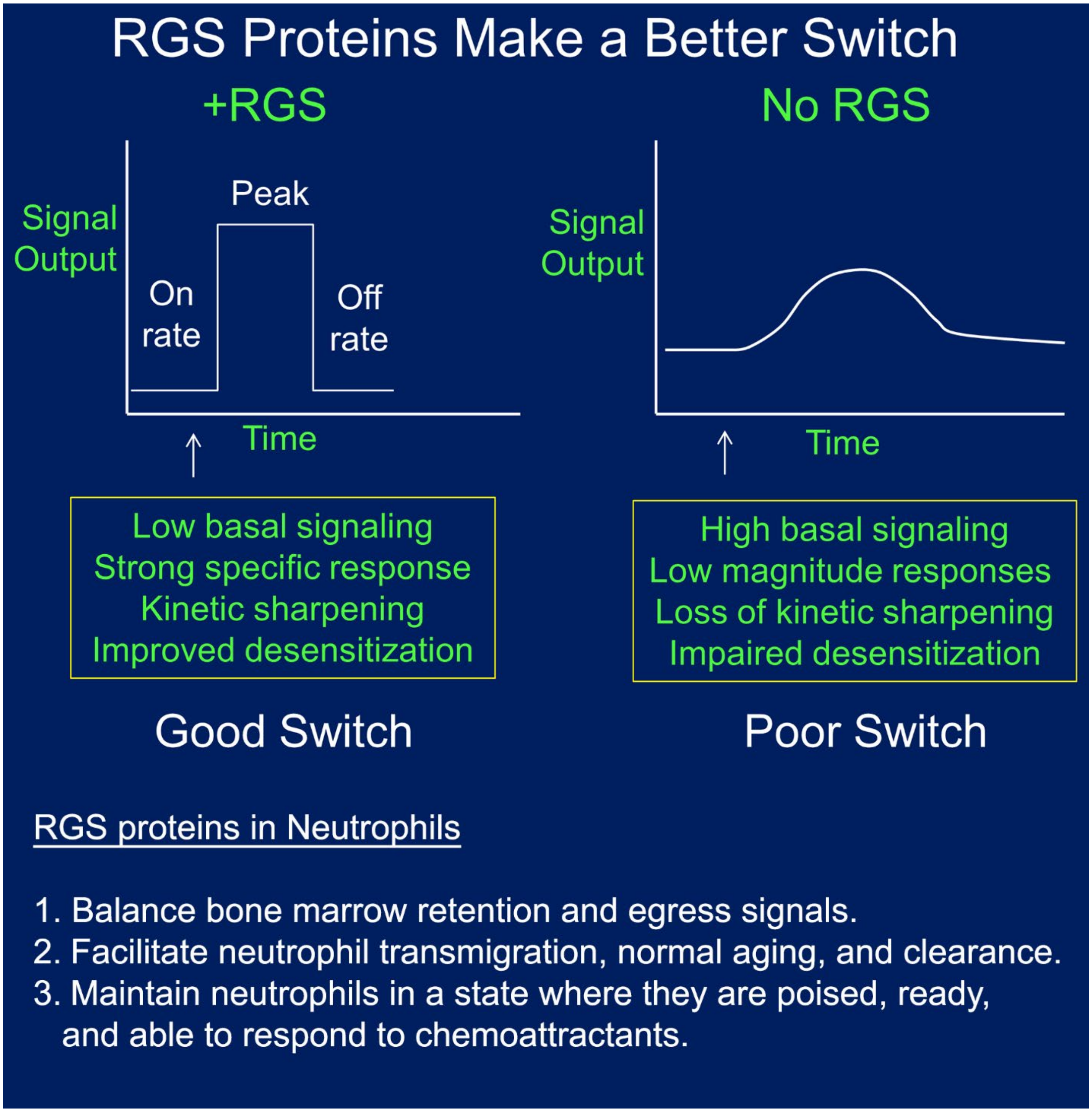

Supplementary Figure 8. Model of RGS protein function in chemoattractant receptor signaling in neutrophils.

Table S1

		BM Immature		BM Mature	
		WT (CD45.1, n=3)	G184S (CD45.2, n=3)	WT (CD45.1, n=3)	G184S (CD45.2, n=3)
CXCR4	extracellular	160.0 ± 0.58	211.0 ± 4.04	108.5 ± 1.44	191.5 ± 0.29
	Intracellular	910.5 ± 59.18	937.0 ± 22.52	961.5 ± 77.65	1130.5 ± 34.35
	Ratio (MFI_{extra}/MFI_{Intra})	0.18 ± 0.012	0.23 ± 0.010	0.11 ± 0.011	0.17 ± 0.005
CXCR2	extracellular	773.5 ± 23.96	*** 524.5 ± 80.54	1940.0 ± 32.33	*** 1641.5 ± 116.91
	Intracellular	358.0 ± 25.40	421.5 ± 75.34	360.5 ± 54.56	826.0 ± 101.04
	Ratio (MFI_{extra}/MFI_{Intra})	2.19 ± 0.224	*** 1.41 ± 0.465	5.68 ± 0.982	*** 2.09 ± 0.406
CD62L	extracellular	2118.5 ± 4.91	*** 1523.5 ± 11.84	2904.5 ± 7.22	2747.5 ± 29.73

Supplementary Table 1. Neutrophil CXCR4, CXCR2, and CD62L Expression Levels on Bone Marrow Neutrophils. Flow cytometry data shows CXCR4, CXCR2, and CD62L expression levels of WT vs G184S BM neutrophils. Neutrophils isolated from bone marrows of 1:1 chimeric mice are further divided into immature and mature subsets. Data are means ± SEMs. *** p <0.0005.

Table S2

		Spleen		Lung		Liver	
		WT (CD45.1, n=3)	G184S (CD45.2, n=3)	WT (CD45.1, n=3)	G184S (CD45.2, n=3)	WT (CD45.1, n=3)	G184S (CD45.2, n=3)
CXCR4	extracellular	163.5 ± 3.18	209.7 ± 4.91	181.5 ± 2.02	150.0 ± 5.20	184.0 ± 13.86	219.0 ± 16.17
	Intracellular	1670.0 ± 12.12	1623.0 ± 32.91	2157.0 ± 55.43	2230.5 ± 77.65	1242.5 ± 21.65	1249.0 ± 113.61
	Ratio (MFI_{extra}/MFI_{Intra})	0.10 ± 0.003	0.13 ± 0.005	0.08 ± 0.001	0.07 ± 0.005	0.15 ± 0.014	0.18 ± 0.030
CXCR2	extracellular	2014.5 ± 21.07	*** 925.5 ± 10.10	1744.5 ± 43.59	*** 699.5 ± 14.15	797.0 ± 6.35	*** 310.5 ± 2.60
	Intracellular	2805.0 ± 20.79	2639.0 ± 36.37	1638.5 ± 63.80	1755.0 ± 12.70	1687.0 ± 15.59	1379.5 ± 7.79
	Ratio (MFI_{extra}/MFI_{Intra})	0.72 ± 0.002	*** 0.35 ± 0.001	1.07 ± 0.068	*** 0.40 ± 0.011	0.47 ± 0.008	*** 0.23 ± 0.003
CD62L	extracellular	842.0 ± 1.16	*** 397.5 ± 7.22	508.0 ± 22.52	*** 338.5 ± 1.44	591.0 ± 2.31	*** 261.5 ± 2.60

Supplementary Table 2. Neutrophil CXCR4, CXCR2, and CD62L Expression Levels on Spleen, Lung, and Liver Neutrophils. Flow cytometry data shows CXCR4, CXCR2, and CD62L expression levels of WT vs G184S BM reconstituted mice. Neutrophils were isolated from spleen, lung, and liver from 1:1 chimeric mice. Data are means ± SEMs. *** p <0.0005.

2. Video Legends

Supplementary Video 1. In Vivo Visualization of WT vs G184S Basal Neutrophil Mobility within Murine Bone Marrow Niches. The multi-photon (MP) intravital microscopy (IVM) movie on the left captures the mobility of WT neutrophils (green) within the skull bone marrow (BM) of a PBS-treated LysM-GFP WT BM reconstituted mouse. The sequence shows a ~60-min period. The MP-IVM movie on the right shows the mobility of G184S neutrophils (green) within the skull BM of a PBS-treated LysM-GFP G184S BM reconstituted mouse. The sequence shows a ~55-min period. Blood vessels are outlined by i.v. injection of Evans Blue (red). Images were captured at ~1 frame per 12 s with ~20X magnification.

Supplementary Video 2. Visualizing WT vs G184S Neutrophil Mobility within Stimulated Murine Bone Marrow Niches *In Vivo.* The MP-IVM movie on the left captures the mobility of WT neutrophils (green) within the skull BM of a CXCL1-treated LysM-GFP WT BM reconstituted mouse. The sequence shows a ~60-min period. The MP-IVM movie on the right shows the mobility of G184S neutrophils (green) within the skull BM of a CXCL1-treated LysM-GFP G184S BM reconstituted mouse. The sequence shows a ~43-min period. Blood vessels are outlined by i.v. injection of Evans Blue (red). Images were captured at ~1 frame per 12 s with ~20X magnification.

Supplementary Video 3. *In Vivo* Visualization of WT vs G184S Neutrophil Trafficking in Inflamed Murine Cremaster Muscle Venules. The confocal IVM movies compared WT (left) and G184S (right) neutrophil trafficking in IL-1β (i.s.) + AMD3100 (i.p.)-treated cremaster muscles with Alexa Fluor 488-anti-Gr1 mAb neutrophils (cyan) and Alexa Fluor 647-anti-CD31 mAb venules (blue). On the left, the movie captures WT neutrophils adhering, crawling, transmigrating through venular walls, and migrating within the interstitial tissue in a WT BM reconstituted mouse. The sequence shows a ~60-min period. The movie on the right shows G184S neutrophils trafficking within and outside the inflamed venules in a G184S BM reconstituted mouse. The sequence shows a ~60-min period. Images were captured at ~1 frame per 30 s with ~25X magnification.

Supplementary Video 4. *In Vivo* WT Neutrophil Transmigration in Inflamed Murine Cremaster Muscle Venules. At a higher magnification, these confocal IVM movies capture Alexa Fluor 488-anti-Gr1 mAb immunostained WT neutrophils (cyan) undergoing transmigration (TEM) in WT BM reconstituted mice treated with IL-1β (i.s.) and AMD3100 (i.p.). Endothelial cells (ECs) of the venule segments (blue) were immunostained *in vivo* with Alexa Fluor 647-anti-CD31 mAb. The left movie shows a WT neutrophil undergoing TEM. The movie taken from the luminal side shows an incoming WT neutrophil adhering to venule at EC junctions, and squeezing through the EC junction via extensive changes in neutrophil cell shape. The yellow track shows the course of WT neutrophil TEM with the appearance and movement of the yellow sphere indicating the start to end of this process. The sequence shows a ~18-min period. The right confocal IVM movie taken from the luminal side captures 2 WT neutrophils undergoing consecutive TEM (yellow and red tracks) through an opened endothelial pore "hot spot" located on a venule segment. The spheres (yellow and red spheres) appearing on the tracks indicate the start to end of each process. The sequence shows a ~17-min period. Images were captured at ~1 frame per 30 s with ~75X magnification.

Supplementary Video 5. Visualizing Intravascular G184S Neutrophil Behaviors and Transmigration *In Vivo.* At a higher magnification, these confocal IVM movies capture the intravascular behaviors and TEM of Alexa Fluor 488-anti-Gr1 mAb immunostained G184S neutrophils (cyan) in G184S BM reconstituted mice treated with IL-1β (i.s.) and AMD3100 (i.p.). The venule segment (blue) are immunostained *in vivo* with Alexa Fluor 647-anti-CD31 mAb. The top left movie shows a G184S neutrophil adhering to the venule segment. The adherent G184S neutrophil eventually dislodged and disappeared into the circulation. The sequence shows a ~18-min period. The top right movie captures an incoming circulating G184S neutrophil adhering to and crawling along the intravascular venular wall, eventually detaching and returns to the

circulation. The sequence shows a ~17-min period. The movie on the bottom left shows an incoming circulating G184S neutrophil adhering to the venule, squeezing through the EC wall via substantial changes in neutrophil cell shape. The sequence shows a ~23-min period. The movie on the bottom right captures an incoming circulating G184S neutrophil adhering to and crawling along the intravascular venular wall, eventually undergo TEM. The sequence shows a ~30-min period. The yellow spheres appearing along the tracks indicate the starting and ending of the described processes. Images were all taken from the luminal side and captured at ~1 frame per 30 s with higher magnification (~60 – 75X).

Supplementary Video 6. *In Vivo* Visualization of WT vs G184S Neutrophil Trafficking in Laser Damaged Murine Inguinal Lymph Nodes. The confocal IVM movies compared WT (left) and G184S (right) neutrophil trafficking within inguinal lymph nodes (LNs) after laser-induced damage (white spot). The LN vasculature (magenta) was visualized with Alexa Fluor 555-anti-CD31 mAb immunostaining, and the Alexa Fluor 488-anti-Gr1 mAb immunostained the WT neutrophils (green) *in vivo*. The left movie captures the result of WT neutrophil trafficking after laser-induced damage within the LN of a WT BM reconstituted mouse. The WT neutrophils accumulate within high endothelial venules (HEVs) near the laser damage site and eventually transmigrate into the LN tissue and travel towards the damage site. The sequence shows a ~60-min period. On the right, the movie shows the G184S neutrophils trafficking within an inguinal LN of a G184S BM reconstituted mouse after laser damage. The sequence shows a ~60-min period. Laser damage was induced inside the LN parenchyma by applying 50% of the MP laser power at zoom 25 for 3 s. Images were taken immediately after laser damage at ~1 frame per 30 s with ~ 25X magnification.

Supplementary Video 7. Visualizing WT vs G184S Neutrophil Trafficking in Inflamed Murine Liver *In Vivo.* The confocal IVM movie on the left shows the result of WT neutrophil trafficking and activity in the liver of a WT BM reconstituted mouse with ConA-induced inflammation. The WT neutrophils (green) and liver sinusoidal vasculature (blue) are immunostained *in vivo* with Alexa Fluor 488-anti-Gr1 mAb and Alexa Fluor 647-anti-CD31 mAb, respectively. The sequence shows a ~40-min period. On the right, the movie captures the result of G184S neutrophil trafficking and activity in the liver of a G184S BM reconstituted mouse with ConA-induced inflammation. The sequence shows a ~40-min period. Images were captured at ~1 frame per 30 s with ~25X magnification.

Supplementary Video 8. *In Vivo* Visualization of WT vs G184S Neutrophil and Platelet Interactions in Inflamed Murine Liver. On the left, the confocal IVM movie shows the result of WT neutrophil (green) and WT platelets (red) immunostained with Alexa Fluor 488-anti-Gr1 mAb and DyLight 649-anti-GP1bβ mAb, respectively, during liver inflammation. The neutrophils and platelets traffic and interact within the Alexa Fluor 555-anti-CD31 mAb immunostained liver sinusoid (violet) of a WT BM reconstituted mouse with ConA-induced inflammation. The adherent neutrophils interact with circulating platelets and platelets aggregates to form clots. The sequence shows a ~30-min period. The right confocal IVM movie captures the result of G184S neutrophil and platelets interacting within the liver sinusoid of a G184S BM reconstituted mouse with ConA-induced inflammation. The platelets interact with adherent neutrophils, formed platelet aggregates, and larger platelet clots within the sinusoid. The sequence shows a ~30-min period. Images were captured at ~1 frame per 30 s with ~50X magnification.

Supplementary Video 9. *In Vivo* Neutrophil Fragmentation and Netosis in Inflamed Murine Liver. The left confocal IVM movie shows the fragmentation of an Alexa Fluor 488-anti-Gr1 mAb immunostained G184S neutrophil (green) within a liver of a G184S BM reconstituted mouse with ConA-induced inflammation. The sinusoid vasculature (blue) are immunostained with Alexa Fluor 647-anti-CD31 mAb. In the middle, the neutrophil crawled along the sinusoid, elongates, and breaks off a small fragment. The sequence shows a ~26-min period. On the right, the confocal IVM movie captures a G184S neutrophil

undergoing netosis within the liver sinusoid during ConA-induced inflammation. The sequence shows a ~40-min period. Images were captured at ~1 frame per 30 s with ~65X magnification.

3. Resource Table

REAGENT or RESOURCE	SOURCE	IDENTIFIER
**Antibodies**		
Anti-mouse CD11b-APC-Cy7 (clone M1/70)	BD Pharmingen	Cat#553312; RRID: AB_396772
Anti-mouse CD11b- Brilliant Violet (BV) 650™ (clone M1/70)	BioLegend	Cat#101259; RRID: AB_2566568
Anti-mouse CD11c-Biotin (clone HL3)	BD Pharmingen	Cat#553800; RRID: AB_395059
Anti-mouse CD11c-BV711™ (clone N418)	BioLegend	Cat#117349; RRID: AB_2563905
Anti-mouse F4/80-Biotin (clone BM8)	BioLegend	Cat#123106; RRID: AB_893501
Anti-mouse F4/80-APC (clone BM8)	BioLegend	Cat#123106; RRID: AB_893481
Anti-mouse Gr-1-Biotin (clone RB6-8C5)	BD Pharmingen	Cat#1088430; RRID: AB_394641
Anti-mouse Ly6C-V450 (clone AL-21)	BD Pharmingen	Cat#560453; RRID: AB_1645284
Anti-mouse Ly6G-PE Cy7 (clone 1A8)	BD Pharmingen	Cat#560601; RRID: AB_1727562
Anti-mouse NK1.1-Biotin (clone PK136)	BD Pharmingen	Cat# 553163; RRID: AB_394675
Anti-mouse NK1.1-APC (clone PK136)	BioLegend	Cat#108710; RRID: AB_313397
Anti-mouse TCRγδ-Biotin (clone GL3)	BD Pharmingen	Cat# 553176; RRID: AB_394687
Anti-mouse TCRγδ-APC (clone GL3)	BioLegend	Cat#118116; RRID: AB_1731813
Anti-mouse B220-Biotin (clone RA3-6B2)	BD Pharmingen	Cat#553086; RRID: AB_394616
Anti-mouse B220-PerCP/Cy5.5 (clone RA3-6B2)	BioLegend	Cat#103236; RRID: AB_893354
Anti-mouse CD117-Biotin (clone 1B8)	BD Pharmingen	Cat# 553353; RRID: AB_394804
Anti-mouse TER-119-Biotin (clone TER-119)	BD Pharmingen	Cat#553672; RRID: AB_394985
Anti-mouse CD3-Biotin (clone 145-2C11)	BioLegend	Cat#100304; RRID: AB_312669
Anti-mouse CD4-Biotin (clone GK1.5)	BioLegend	Cat#100404; RRID: AB_312689
Anti-mouse CD5-PerCP Cy5.5 (clone 53-7.3)	BioLegend	Cat#100624; RRID: AB_2563433
Anti-mouse CD8-Biotin (clone 53-6.7)	BioLegend	Cat#100704; RRID: AB_312743
Anti-mouse CD62L-PE (clone Mel-16)	BioLegend	Cat#104408; RRID: AB_313095
Anti-mouse CD45.1-FITC (clone A20)	BioLegend	Cat#110706; RRID: AB_313495
Anti-mouse CD45.1-PE (clone A20)	BioLegend	Cat#110708; RRID: AB_313497
Anti-mouse CD45.2-APC (clone 104)	BioLegend	Cat#109814; RRID: AB_389211
Anti-mouse CD45.2-Alexa Fluor® 488 (clone 104)	BioLegend	Cat#109816; RRID: AB_492848
Anti-mouse CD182-PE (CXCR2, clone SA045E1)	BioLegend	Cat#149610; RRID: AB_2565690
Anti-mouse CD184-PE (CXCR4, clone 2B11)	Thermo Fisher Scientific	Cat#12-9991-82; RRID: AB_891391
Anti-mouse CD169-A488 (clone 3D6.112)	BioLegend	Cat#142419; RRID: AB_2566436
Anti-mouse Ly6G-A594 (clone1A8)	BioLegend	Cat#127636; RRID: AB_2563207
Anti-mouse CD31-APC (clone 390)	eBioscience	Cat#17-0311-82; RRID: AB_657735

Anti-mouse Ly6G-FITC (clone 1A8)	eBioscience	Cat#11-9668-82; RRID:AB_2572532
Anti-mouse CD45-BV650™ (clone 30-F11)	BioLegend	Cat#103151; RRID: AB_2565884
Anti-mouse CD45-BV785™ (clone 30-F11)	BioLegend	Cat#103249; RRID: AB_2564590
Anti-mouse CD31, purified (clone 390)	BioLegend	Cat#102402; RRID: AB_312897
Anti-mouse Gr-1, purified (clone RB6-8C5)	BioLegend	Cat#108402; RRID: AB_313367
Anti-GPIbβ-DyLight649 (derivatized)	Emfret Analytics	Cat#X649; RRID: AB_2861336
Streptavidin-BV605™	BioLegend	Cat#405229
Streptavidin-APC	BioLegend	Cat#405207
Phospho-Ezrin (Thr567)/Radixin (Thr564)/Moesin (Thr558) (48G2) Rabbit mAb	Cell signaling	Cat#3726S; RRID:AB_10560513
Alexa Fluor™ 488 Phalloidin	Thermo Fisher	Cat# A12379; RRID:AB_2315147
Alexa Fluor™ 647 Phalloidin	Thermo Fisher	Cat#A22287; RRID:AB_2620155
Isotype Control Rat IgG2b, kappa-PE (clone eB149/10H5)	Thermo Fisher	Cat#12403182; RRID: AB_470042
Isotype Control Rat IgG2a, kappa-PE (clone RTK2758)	BioLegend	Cat#400508; RRID: AB_326530
Rabbit IgG Monoclonal Antibody; Isotype Control, Alexa Fluor 647 Conjugated, Clone DA1E	Cell signaling	Cat#2985S; RRID:AB_1196589
F(ab')2-Goat anti-Rabbit IgG (H+L) Cross-Adsorbed Secondary Antibody, Alexa Fluor 647	Thermo Fisher	Cat#A21246; RRID: AB_2535814
Isotype Control Rat IgG2a, kappa-FITC (clone MOPC-173)	BioLegend	Cat#400208; RRID: AB_2884007
Isotype Control Rat IgG2a, kappa-Alexa Fluor® 488 (clone MOPC-173)	BioLegend	Cat#400208; RRID: AB_2864283
Isotype Control Rat IgG2b, kappa-PE-Cy7 (clone RTK4530)	BioLegend	Cat#400618; RRID: :AB_326560
Isotype Control Rat IgG2b, kappa-Alexa Fluor® 647 (clone RTK4530)	BioLegend	Cat#400626; RRID: :AB_389343
Isotype Control Rat IgG2b, kappa-FITC (clone RTK4530)	BioLegend	Cat#400605; RRID: :AB_326549
**Chemicals, Peptides, and Recombinant Proteins**		
Sphingosine-1-phosphate (d18:1)	Cayman	Cat#62570-1MG
fMLP (N-Formyl-Met-Leu-Phe)	Tocoris	Cat#1921-1MG
Recombinant murine Complement Component C5a	R&D Systems	Cat#2150-C5-025
Recombinant murine CXCL1 (KC)	R&D Systems	Cat#453-KC
Recombinant murine CXCL2	PeproTech	Cat#250-15
Recombinant murine CXCL12	PeproTech	Cat#250-20A
Bovine Serum albumin fatty acid free (low endotoxin)	Sigma-Aldrich	Cat#8806
AMD 3100	Sigma-Aldrich	Cat# A5602-5MG
KRH 3955	Tocris	Cat#6126-10MG

PMA	Sigma-Aldrich	Cat#P8139
Helix NP™ NIR	BioLegend	Cat#425301
DAPI	BioLegend	Cat#422801
Avertin (Tribromoethanol)	Sigma-Aldrich	Cat#T48402-5G
Evans Blue Dye	Sigma-Aldrich	Cat#E2129
Recombinant murine ICAM-1-FC	R&D Systems	Cat#796-IC-050
Recombinant murine IL-1β	R&D Systems	Cat#401-ML-005
Concanavalin A (ConA)	InvivoGen	Cat#inh-cona
eBioscience™ Intracellular Fixation & Permeabilization Buffer Set	ThermoFisher	Cat# 88-8824-00
FLIPR Calcium 4 Assay kit	Molecular Devices	Cat# R8142
**Critical Commercial Assays**		
Alexa Fluor 488 protein labeling kit	Thermo Fisher Scientific	Cat#A10235
Alexa Fluor 555 protein labeling kit	Thermo Fisher Scientific	Cat#A20174
Alexa Fluor 568 protein labeling kit	Thermo Fisher Scientific	Cat#A10238
Alexa Fluor 647 protein labeling kit	Thermo Fisher Scientific	Cat#A20173
FLIPR Calcium 4 assay kit	Molecular Devices	Cat# R8142
LIVE/DEAD™ Fixable aqua dead cell stain kit	Thermo Fisher Scientific	Cat#L34966
LIVE/DEAD™ Fixable yellow dead cell stain kit	Thermo Fisher Scientific	Cat#L34968
LIVE/DEAD™ Fixable Near-IR dead stain kit	Thermo Fisher Scientific	Cat#L34976
APC BrdU Flow kit	BD Biosciences	Cat#552598
Anti-Ly-6G MicroBeads UltraPure, mouse	Miltenyi Biotec	Cat#130-120-337
**Software and Algorithms**		
SOFT max Pro 5.2	Molecular Devices	https://www.moleculardevices.com/
FlowJo v 9.9	Tree Star	https://www.flowjo.com/
FlowJo v10.7	Tree Star	https://www.flowjo.com/
ImageJ 1.53a	NIH	https://imagej.nih.gov/ij/
Imaris v9.3.1	Bitplane	http://www.bitplane.com/imaris
Prism v8.0.1	GraphPad	https://www.graphpad.com/scientific-software/prism/

4. Flow cytometer information.

	Laser lines	Emission filters	Fluorochrome
MACS Quant	488 nm	525/50 nm	FITC, Alexa Fluor 488
		585/40 nm	PE
		655–730 nm	PerCP Cy5.5
		750 nm LP	PE-Cy7
	640 nm	655–730 nm	APC
		750 nm LP	APC Cy7, LIVE DeadLIVE/DEAD Fixable Near-IR stain
	405 nm	450/50 nm	pacific Blue, BV-421, Horizon V450, efluor 450

	Laser lines	Emission filters	Fluorochrome
FACS CANTOII	488 nm	530/30 nm	FITC
		585/42 nm	PE
		670 LP	PerCP Cy5.5
		780/60 nm	PE-Cy7
	633 nm	660/20 nm	APC
		780/60 nm	APC Cy7, LIVE DeadLIVE/DEAD Fixable Near-IR stain
	405 nm	450/50 nm	pacific Blue, BV-421, Horizon V450, efluor 450
		502-525 nm	LIVE/Dead Fixable Aqua stain

	Laser lines	Emission filters	Fluorochrome
FACSCelesta SORP	488 nm	530/30 nm	FITC
		710/50 nm	PerCP Cy5.5
	561 nm	586/15	PE
		610/20	PE CF594
		670/30	PE-Cy5
		780/60 nm	PE-Cy7
	640 nm	660/20 nm	APC
		730/45	Alexa Fluor 700, APC 700
		780/60 nm	APC Cy7, LIVE/DEAD Fixable Near-IR stain
	405 nm	450/50 nm	pacific Blue, BV-421, Horizon V450, efluor 450
		525/50 nm	LIVE/Dead Fixable Aqua stain
		610/20	BV605, LIVE/Dead Fixable Yellow stain
		670/30	BV650
		710/50	BV711
		780/60	BV786
